# D-Allulose Reduces Hypertrophy and Endoplasmic Reticulum Stress Induced by Palmitic Acid in Murine 3T3-L1 Adipocytes

**DOI:** 10.3390/ijms25074059

**Published:** 2024-04-05

**Authors:** Maria Sofia Molonia, Federica Lina Salamone, Antonio Speciale, Antonella Saija, Francesco Cimino

**Affiliations:** 1 Department of Chemical, Biological, Pharmaceutical and Environmental Sciences, University of Messina, Viale F. Stagno D’Alcontres 31, 98166 Messina, Italy; mmolonia@unime.it (M.S.M.); federica.salamone@studenti.unime.it (F.L.S.); asaija@unime.it (A.S.); fcimino@unime.it (F.C.); 2 “Prof. Antonio Imbesi” Foundation, University of Messina, 98100 Messina, Italy

**Keywords:** D-allulose, rare sugars, natural sweeteners, adipocyte hypertrophy, obesity

## Abstract

Natural rare sugars are an alternative category of sweeteners with positive physiologic and metabolic effects both in in vitro and animal models. D-allulose is a D-fructose epimer that combines 70% sucrose sweetness with the advantage of an extremely low energy content. However, there are no data about the effect of D-allulose against adipose dysfunction; thus, it remains to be confirmed whether D-allulose is useful in the prevention and in treatment of adipose tissue alterations. With this aim, we evaluated D-allulose’s preventive effects on lipid accumulation in 3T3-L1 murine adipocytes exposed to palmitic acid (PA), a trigger for hypertrophic adipocytes. D-allulose in place of glucose prevented adipocyte hypertrophy and the activation of adipogenic markers C/EBP-β and PPARγ induced by high PA concentrations. Additionally, D-allulose pretreatment inhibited the NF-κB pathway and endoplasmic reticulum stress caused by PA, through activation of the Nrf2 pathway. Interestingly, these effects were also observed as D-allulose post PA treatment. Although our data need to be confirmed through in vivo models, our findings suggest that incorporating D-allulose as a glucose substitute in the diet might have a protective role in adipocyte function and support a unique mechanism of action in this sugar as a preventive or therapeutic compound against PA lipotoxicity through the modulation of pathways connected to lipid transport and metabolism.

## 1. Introduction

An increase in the occurrence of obesity has become a major health problem in adults, as well as among children and adolescents. It is defined as an excess of corporal adiposity characterized by body weight gain. Lipid accumulation is correlated with insulin resistance and cardiovascular events, with a large decrease in life expectancy and increased mortality. Obesity is due to an imbalance in systemic energy homeostasis and is associated with a sedentary lifestyle with an excess consumption of highly processed, energy-dense food that has a poor nutritional value [1,2]. Excessive energy intake over expenditure leads, in fact, to an energy imbalance, which determines the dysfunctional growth of adipose tissue, resulting in an increase in the number (hyperplasia) and size (hypertrophy) of adipocytes, to counteract the need for improved lipid storage [3]. Excessive lipid accumulation in adipose tissue is closely related to significant alterations in proinflammatory cytokines serum concentrations, determining a condition defined as “chronic low-grade inflammation”, which seems to have an important pathophysiological role in the development of several metabolic diseases related to obesity, such as dyslipidemias, mitochondrial dysfunction, insulin resistance, and hyperglycemia [4,5]. The lipotoxicity condition in adipose tissue can also determine very significant alterations in the biophysical properties of cellular organelles [6]. In this regard, alterations affecting the endoplasmic reticulum (ER), one of the main sites for lipid biosynthesis, appear to be closely associated with an overload of saturated fatty acids and cholesterol: therefore, changes in the phospholipid composition of ER membrane occur, resulting in the onset of ER stress, the accumulation of misfolded proteins within the lumen, and cell death by apoptosis [7].

Recently, major concerns have been expressed regarding the intake of metabolizable free sugars, which are usually replaced by artificial high-intensity sweeteners, such as aspartame, acesulfame potassium, saccharin, sucralose, and others, approved as food additives by the European Food Safety Authority (EFSA) and U.S. Food and Drug Administration (FDA) [8,9]. However, these alternative sweeteners are associated with unfavorable effects on health, such as glucose intolerance and failure to cause weight reduction [10]. Interestingly, naturally occurring rare sugars have recently appeared as a valuable alternative category of sweeteners. Rare sugars (such as D-allulose, D-tagatose, D-sorbose, and D-allose) are monosaccharides and disaccharides occurring in small quantities in nature, with over 40 different types of compounds identified and characterized by slight differences in their chemical structure compared with traditional sugars. The consumption of rare sugars as a natural alternative sweetener has shown numerous positive physiologic and metabolic effects, such as improved glycemic response and weight loss both in in vitro and animal models [11]. However, the biological functions and molecular mechanisms of rare sugars have rarely been reported.

D-allulose (also known as D-psicose) is an epimer of D-fructose (at the C-3 position), bearing 70% of the sweetness of sucrose, with the advantage of an ultra-low dietary energy content (0.4 kcal/g). D-allulose provides a taste, performance, and texture similar to other sugars, but it is predominantly preferred for its exceptionally low-calorie value, probably related to its high elimination rate in the unmodified form (95% in the urine within 7 h after intravenous injection in rats) [12]. It has been demonstrated, in fact, that D-allulose, in contrast to other sugars that are metabolized for energy, is absorbed by the small intestine and eliminated in the urine without undergoing significant metabolic changes [13]. D-allulose has exhibited, in this regard, exceptional stability in simulated gastric fluid, in fasted-state simulated intestinal fluid, and in both human and rat hepatocytes [14]. Furthermore, it is not involved in glucose-related metabolic processes, leading to an inability to contribute to the production of hepatic energy [15].

D-allulose has been approved as GRAS (Generally Recognized As Safe) by the FDA and is allowed to be excluded from total and added sugar counts on food labels. Acute and longer-term randomized controlled trials have evaluated D-allulose’s influence on plasma glucose and insulin release both in healthy and type-2-diabetic subjects, showing benefits in both populations [11]. However, these studies focused on a limited range of outcomes (e.g., body weight, long- or short-term glycemic markers), and the biological mechanisms of these outcomes are not yet clear. Additionally, no in vivo study has reported the effects of this rare sugar on healthy people as being preventive of weight gain. Recently, Lee et al. [16] demonstrated the anti-adipogenic effects of D-allulose in 3T3-L1 adipocytes. Similarly, since a reduction in sugar intake may require sugar substitutes, Moon et al. [17] investigated the effect of a 50% substitution of glucose with D-allulose on 3T3-L1 adipocytes and found that D-allulose could suppress adipocyte differentiation and lipid accumulation through modulating adipogenic transcription factors. However, there are no data regarding the effect of D-allulose against adipose dysfunction, which is caused by excessive fat storage within adipose tissue. Furthermore, it remains to be confirmed whether the use of D-allulose can be useful in the prevention and treatment of adipose tissue alterations.

In our study, using a simple and validated in vitro experimental model, we evaluated the preventive effects of D-allulose on lipid accumulation in murine adipocytes exposed to palmitic acid (PA), a saturated fatty acid able to produce adipocyte hypertrophy. Under our experimental conditions, fully differentiated 3T3-L1 adipocytes were pretreated with D-allulose, which was added to the medium at different concentrations in substitution of the same amount of glucose, and then exposed to PA, with the aim of investigating the main molecular pathways involved in adipogenesis, the inflammatory process, and ER stress. Additionally, the same pathways were evaluated for cells treated with D-allulose after exposure to PA in order to also evaluate the potential therapeutic effects of this rare sugar.

## 2. Results

### 2.1. D-Allulose Protects against PA-Induced Hypertrophy

**Figure 1:**
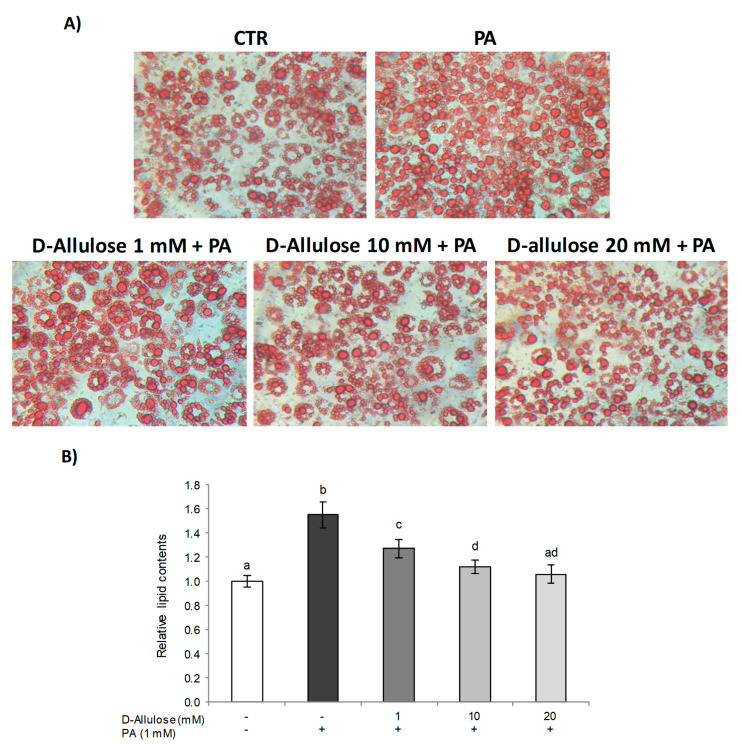
D-allulose effect on PA-induced lipid accumulation. Totally differentiated 3T3-L1 cells were cultured for 24 h in a medium containing D-allulose (1–10–20 mM) and exposed for 24 h to 1 mM PA. Cells treated with the PA vehicle alone were used as controls. (**A**) Staining with Oil Red O (original magnification at 40×). Representative images of three independent experiments. (**B**) Lipids were extracted with isopropanol and expressed as relative content vs. controls. All data are expressed as mean ± SD of three independent experiments (*n* = 3), each performed in triplicate. Means with the same letter are not significantly different from each other (*p* > 0.05).

It is widely known that in obesity, high free fatty acid (FFA) levels determine a hypertrophic expansion of the adipose tissue, with a consequent increase in the incidence of several metabolic complications [1]. Thus, to examine the effects of D-allulose against PA-induced hypertrophy in 3T3-L1 adipocytes, the accumulation of intracellular fat was first evaluated via the histological technique of Oil Red O staining (Figure 1). The results obtained showed that treatment with high concentrations of PA (1 mM) for 24 h causes a significant increase in lipid droplets compared to control cells, thus demonstrating the lipotoxic state induced by PA in adipose tissue. The pretreatment with D-allulose, at all tested concentrations (1–10–20 mM), conversely, in a dose-dependent way, reduced the hypertrophic state triggered by PA. Treatment with D-allulose alone did not show, however, any significant change compared to the control cells (Figure S1).

Subsequently, in order to assess the mechanism through which D-allulose is able to prevent the accumulation of intracellular lipids, thus reducing the hypertrophy condition in adipose tissue, we investigated the main markers involved in adipogenic differentiation. In particular, during the early stages of differentiation, the activation of CCAAT enhancer binding protein beta (C/EBP-β) occurs, determining the expression of peroxisome proliferator-activated receptor gamma (PPARγ), considered the master regulator of the adipogenesis process [18,19], thus starting the differentiation process. Subsequently, these factors cooperate to determine the complete adipocyte differentiation by the expression of genes typical of mature adipocytes, such as fatty-acid-binding protein 4 (*FABP4*) and fatty acid synthase (*FASN*) [18].

**Figure 2:**
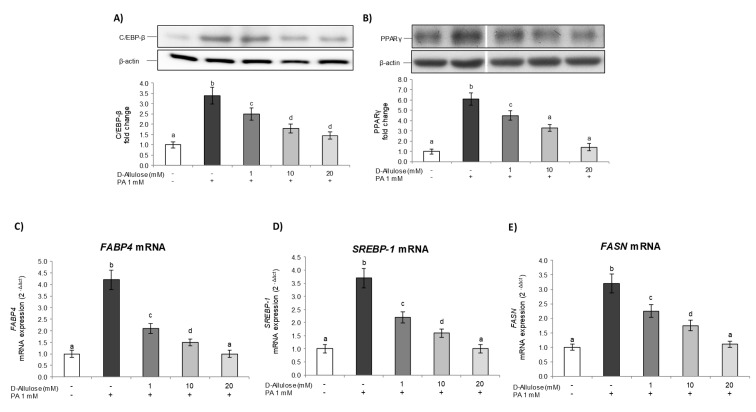
D-allulose’s effect on adipogenesis markers. Totally differentiated 3T3-L1 cells were cultured for 24 h in a medium containing D-allulose (1–10–20 mM) and exposed for 24 h to 1 mM PA. Cells treated with the PA vehicle alone were used as controls. (**A**,**B**) C/EBP-β and PPARγ protein levels; the densitometry results are reported as fold change compared to controls, and the intensity values are normalized to the corresponding value of β-actin. In (**B**), bands are cropped from original Western blot images for illustrative purposes; the uncropped images are available in the Supplementary Material. (**C**–**E**) *FABP4*, *SREBP-1*, and *FASN* mRNA expression was analyzed via real-time PCR, and data are expressed as 2^−ΔΔCt^ and normalized to controls; 18S rRNA was used as housekeeping gene. All data are expressed as mean ± SD of three independent experiments (*n* = 3), each performed in triplicate. Means with the same letter are not significantly different from each other (*p* > 0.05).

First, Western blot analysis was employed to investigate D-allulose’s effects on the expression of C/EBP-β (Figure 2A) and PPARγ (Figure 2B) induced by PA. The results obtained confirm that PA exposure determines a strong enhancement in C/EBP-β and PPARγ levels compared to untreated control cells. Conversely, pretreatment with D-allulose, in a dose-dependent way, reduced the PA effect, restoring the protein levels of C/EBP-β and PPARγ to those of the control cells. Also in this case, treatment with D-allulose alone did not induce any change with respect to basal levels; moreover, low/very low concentrations of glucose, corresponding to the glucose amount present in the treatments with D-allulose, do not affect PPARγ expression (Figure S2). 

Subsequently, to further confirm the observed effects, we also examined, via real-time PCR, the expression levels of *FABP4* (Figure 2C), Sterol regulatory element-binding protein 1 (*SREBP-1*) (Figure 2D), and *FASN* (Figure 2E), downstream genes of the PPARγ pathway involved in fatty acid uptake and lipid accumulation in adipose tissue [20]. The data obtained show a marked increase in *FABP4*, *SREBP-1*, and *FASN* gene expression in response to PA, whereas D-allulose pretreatment dose-dependently reduced these mRNA levels with values comparable to those of the control cells for the highest concentration of D-allulose used (20 mM).

These results thus demonstrate how the replacement of glucose in the medium with D-allulose prevents the hypertrophic condition induced by PA in adipose tissue, modulating the expression of some of the most important markers involved in the adipogenesis process and therefore reducing excessive lipid accumulation in adipose tissue.

### 2.2. D-Allulose Prevents NF-kB Pathway Activation Induced by PA in Adipocytes

Recent evidence has demonstrated how the hypertrophic adipocyte causes very significant functional alterations in adipose tissue, with a consequent increase in the expression and secretion of adipokines with pro-inflammatory action, able to cause a systemic state of low-grade chronic inflammation, to worsen insulin sensitivity, and to contribute to the development of metabolic and cardiovascular complications associated with obesity [21].

Therefore, in order to study the D-allulose effect on PA-induced low-grade inflammation in adipose tissue, we examined the activation of the NF-κB inflammatory pathway, the most important pathway involved in the inflammatory process.

Under homeostatic conditions, in fact, NF-ᴋB is located in the cytoplasm, in an inactive form, linked to its high affinity inhibitor IᴋB. In the presence of activating stimuli, instead, a signal cascade is induced with a consequent dissociation from IᴋB and nuclear translocation, where it determines the expression of genes coding for proinflammatory proteins [22].

**Figure 3:**
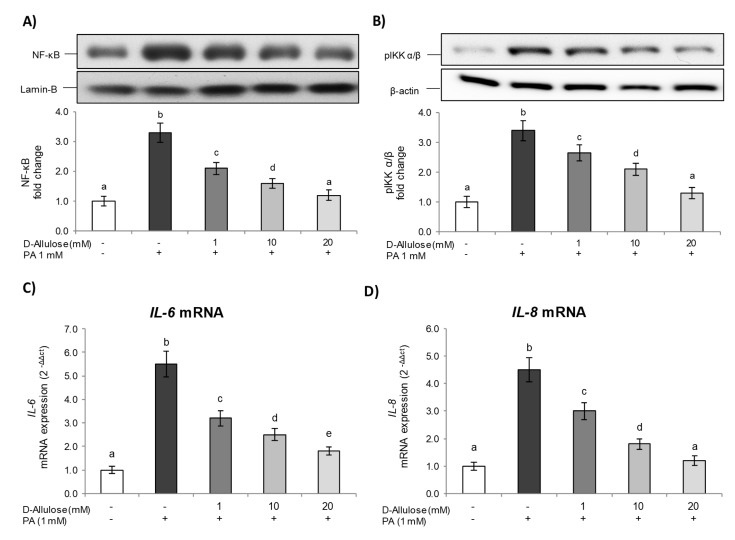
D-allulose effect on NF-κB pathway. Totally differentiated 3T3-L1 cells were cultured for 24 h in a medium containing D-allulose (1–10–20 mM) and exposed for 24 h to 1 mM PA. Cells treated with the PA vehicle alone were used as controls. (**A**,**B**) Nuclear NF-κB and cytoplasmatic pIKK α/β protein levels—the densitometry results are reported as fold change compared to controls, and the intensity values of NF-κB and pIKK α/β proteins were normalized to the corresponding value of lamin-B and β-actin, respectively. (**C**,**D**) *IL-6* and *IL-8* mRNA expression was analyzed via real-time PCR, and data are expressed as 2^−ΔΔCt^ and normalized to controls; 18S rRNA was used as housekeeping gene. All data are expressed as mean ± SD of three independent experiments (*n* = 3), each performed in triplicate. Means with the same letter are not significantly different from each other (*p* > 0.05).

The results reported in Figure 3A show how exposure to PA determines the activation of NF-ᴋB in 3T3-L1 cells, as demonstrated by higher p65 nuclear levels compared to the control. Conversely, the D-allulose pretreatment was able to significantly reduce, starting from the lowest tested concentration (1 mM), the PA effect on nuclear NF-κB levels in a dose-dependent way.

Subsequently, the mechanism responsible for D-allulose’s effects on NF-κB inhibition was further verified by evaluating the cytoplasmic levels of phosphorylated IKK α/β, the main NF-κB activators.

As shown in Figure 3B, in our experimental conditions, PA exposure was able to activate (phosphorylate) IKK α/β. Conversely, D-allulose pretreatment dose-dependently inhibited the IKK α/β phosphorylation induced by PA, thus leading to the NF-κB pathway inhibition shown previously.

Additionally, in order to confirm the transcriptional activity of NF-κB, *IL-6*, and *IL-8* gene expression was evaluated via real-time PCR. These represent, in fact, some of the main cytokines released following an inflammatory stimulus and are able to regulate several aspects of metabolism, including lipolysis, energy expenditure, and glucose disposal in adipose tissue [23].The results obtained also show how, in this case, exposure to PA determines a significant increase in *IL-6* and *IL-8* compared to the control, while pretreatment with D-allulose, at all tested concentrations, was able to reduce the levels of these important pro-inflammatory cytokines in a statistically significant way (Figure 3C,D). Treatment with D-allulose alone did not show any effect with respect to basal levels (Figure S3).

### 2.3. D-Allulose Protects against PA-Induced Oxidative Stress and Activates the Nrf2 Pathway in Hypertrophic Adipocytes

It is widely known that low-grade inflammation in adipose tissue is strongly associated with oxidative stress enhancement [24]. The increase in proinflammatory adipocytokine and chemokine (i.e., TNF-α, IL-1β, MCP-1) production from adipose tissue determines, in fact, lower antioxidant defenses, resulting in ROS overproduction and systemic oxidative stress [25].

Several studies have also shown that elevated ROS levels could trigger many cellular signaling pathways, inducing damage to DNA, proteins, and cells, and lead to the development of several metabolic diseases [26]. Based on these considerations, given the D-allulose ability to reduce the inflammatory condition induced by PA in hypertrophic adipocytes, we examined its ability to influence oxidative stress by evaluating intracellular ROS levels.

**Figure 4:**
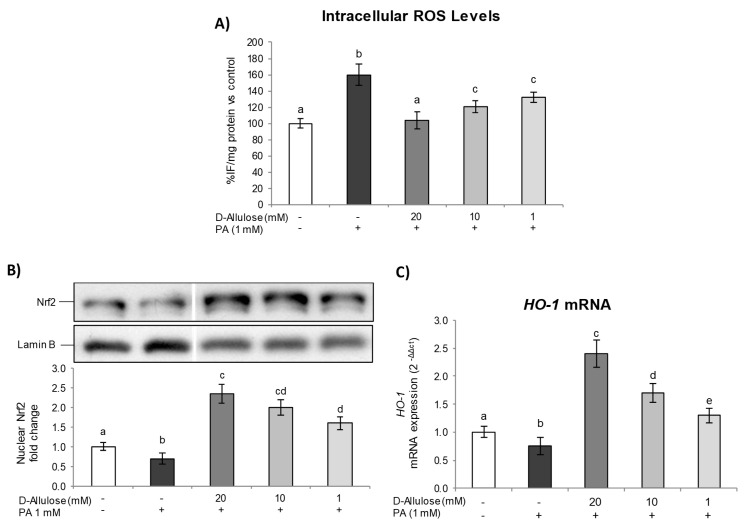
D-allulose’s effect on intracellular ROS level generation and Nrf2 pathway activation. Totally differentiated 3T3-L1 cells were cultured for 24 h in a medium containing D-allulose (1–10–20 mM) and exposed for 24 h to 1 mM PA. Cells treated with the PA vehicle alone were used as controls. (**A**) Intracellular ROS levels; the results are reported as % change in DCF fluorescence intensity/mg of proteins against control. (**B**) Modulation of Nrf2 nuclear levels; the densitometry results are reported as fold change compared to controls, and the intensity value of Nrf2 was normalized to the corresponding value of lamin-B. Bands were cropped from original Western blot image for illustration purposes; the uncropped images are available in the Supplementary Material. (**C**) *HO-1* mRNA levels; data are expressed as 2^−ΔΔCt^ and normalized to controls; 18S rRNA was used as housekeeping gene. All data are expressed as mean ± SD of three independent experiments (*n* = 3), each performed in triplicate. Means with the same letter are not significantly different from each other (*p* > 0.05).

The results reported in Figure 4A show how the lipotoxicity state induced by PA determined a significant increase in ROS levels compared to those of the control cells. On the other hand, pretreatment with D-allulose has been able to significantly reduce intracellular ROS levels starting from 1 mM, restoring them to basal conditions with the highest concentration tested. D-allulose alone did not affect ROS basal levels (Figure S4A). 

Subsequently, in order to determine the potential mechanism underlying D-allulose’s protective effects, we evaluated the nuclear translocation of Nrf2, an important transcription factor with the ability to regulate the expression of antioxidant and detoxifying genes, thereby influencing the maintenance of homeostasis and redox balance in several cells and tissues [27]. Under normal conditions, Nrf2 is inactive, sequestered in the cytoplasm by the inhibitory protein Keap-1. The action of stressful agents causes, instead, the cleavage of Nrf2/Keap-1, resulting in the nuclear translocation of Nrf2 and its subsequent association with the antioxidant responsive element (ARE) sequence, activating the transcription of genes encoding for phase II detoxifying and antioxidant proteins [28].

The results obtained show how the PA exposition results in a significant inhibition of Nrf2 activation/translocation compared to control cells. In contrast, pretreatment with D-allulose, in a dose-dependent way, was able to significantly increase the Nrf2 nuclear levels both in cells exposed and not exposed to PA (Figure 4B and Figure S4B, respectively). Furthermore, we assessed the gene expression of heme oxygenase-1 (*HO-1*), a widely recognized antioxidant enzyme that reflects Nrf2 transcriptional activity, to confirm D-allulose’s ability to trigger an antioxidant adaptive response. As reported in Figure 4C, the lipotoxicity state, induced by PA treatment, determines a significative reduction in *HO-1* basal levels. Conversely, the D-allulose pretreatment, at all tested concentrations, results in an increase in *HO-1* mRNA expression, both in the PA-treated cells and in the unexposed ones (Figure S4C). In summary, therefore, these findings provide evidence that the activation of the Nrf2 pathway contributes to D-allulose’s protective effect against a PA-induced lipotoxicity state in adipose tissue.

### 2.4. D-Allulose Prevents ER Stress in Hypertrophic Adipocytes 

A growing body of evidence supports the theory that oxidative stress and ER stress are interconnected [26]. Alterations to the intracellular redox state can determine, in fact, many changes in the protein folding mechanism and enhance the production of misfolded proteins, causing, consequently, ER stress. Therefore, to resolve this condition, cells activate an intrinsic adaptive mechanism called the “unfolded protein response” (UPR), which triggers several pathways [PERK (PKR-like ER kinase), IRE1 (inositol-requiring enzyme 1), and ATF6 (activating transcription factor 6)], aimed to remove misfolded proteins and to improve the protein folding mechanism, in order to maintain ER homeostasis [29].

**Figure 5:**
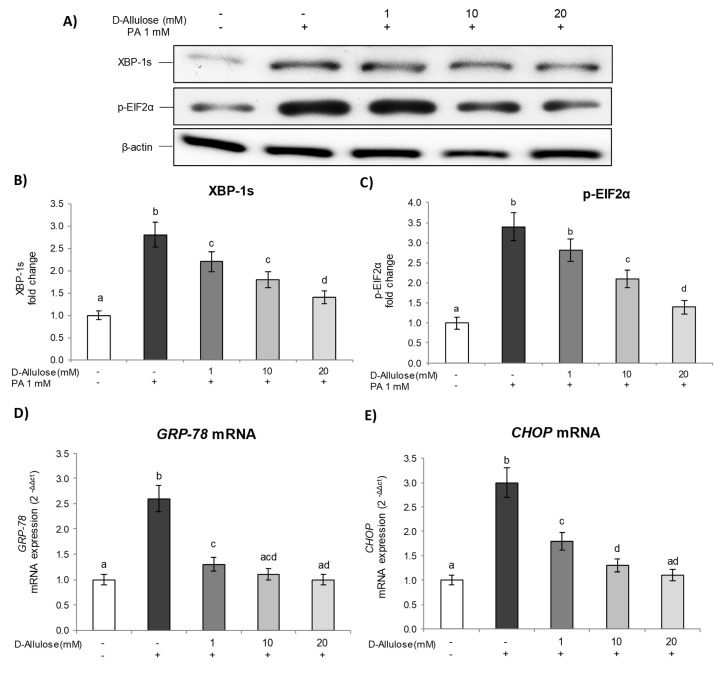
D-allulose’s effects on PA-induced ER stress. Totally differentiated 3T3-L1 cells were cultured for 24 h in a medium containing D-allulose (1–10–20 mM) and exposed for 24 h to 1 mM PA. Cells treated with the PA vehicle alone were used as controls. XBP-1s (**A**,**B**) and p-EIF2α (**A**,**C**) protein levels; the densitometry results are reported as fold change compared to CTR, and the intensity values were normalized to the corresponding value of β-actin. (**D**) *GRP-78* and (**E**) *CHOP* mRNA expression was analyzed via real-time PCR, and data are expressed as 2^−ΔΔCt^ and normalized to controls; 18S rRNA was used as housekeeping gene. All data are expressed as mean ± SD of three independent experiments (*n* = 3), each performed in triplicate. Means with the same letter are not significantly different from each other (*p* > 0.05).

In our experimental conditions, PA determined ER stress, inducing XBP-1s and p-EIF2α expression, compared to control cells, demonstrating the activation of the transcription of genes related to UPR in the presence of a lipotoxicity state, aiming to correct the misfolding protein accumulation within the ER lumen. Conversely, D-allulose pretreatment significantly and dose-dependently inhibited the activation of XBP-1s (Figure 5A,B) and p-EIF2α (Figure 5A–C) induced by PA, thus demonstrating the attenuation of the UPR response.

Subsequently, in order to confirm and further investigate the molecular mechanisms underlying UPR signaling pathway activation, we evaluated the gene expression levels of Glucose Regulated Protein 78 (*GRP78*) and CCAAT enhancer binding protein (C/EBP) homologous protein (*CHOP*), two of the main markers involved in this process. *GRP78*, in fact, is a chaperone molecule involved in the folding of proteins in the endoplasmic reticulum, while *CHOP* is responsible for the apoptotic response mediated by ER stress [30,31]. The results demonstrate how, under our experimental conditions, PA exposure induced *GRP78* and *CHOP* gene expression, compared to controls, thus also confirming the activation of signaling events involved in the UPR response at a transcriptional level. D-allulose pretreatment, on the other hand, was able to significantly reduce the mRNA levels of *GRP78* (Figure 5D) and *CHOP* (Figure 5E), induced by PA, starting from the lowest concentration tested (1 mM). Additionally, treatment with D-allulose alone did not show any change with respect to the basal levels of the ER-stress markers; moreover, low/very low concentrations of glucose, corresponding to the glucose amount present in the treatments with D-allulose, do not affect p-EIF2α levels (Figure S5). These data, therefore, further confirm the ability of D-allulose to reduce the ER stress caused by a lipotoxicity state induced in adipose tissue, through the inhibition of signaling pathways characteristic of the UPR response.

### 2.5. D-Allulose Ameliorates Adipocyte Dysfunction Induced by PA

**Figure 6:**
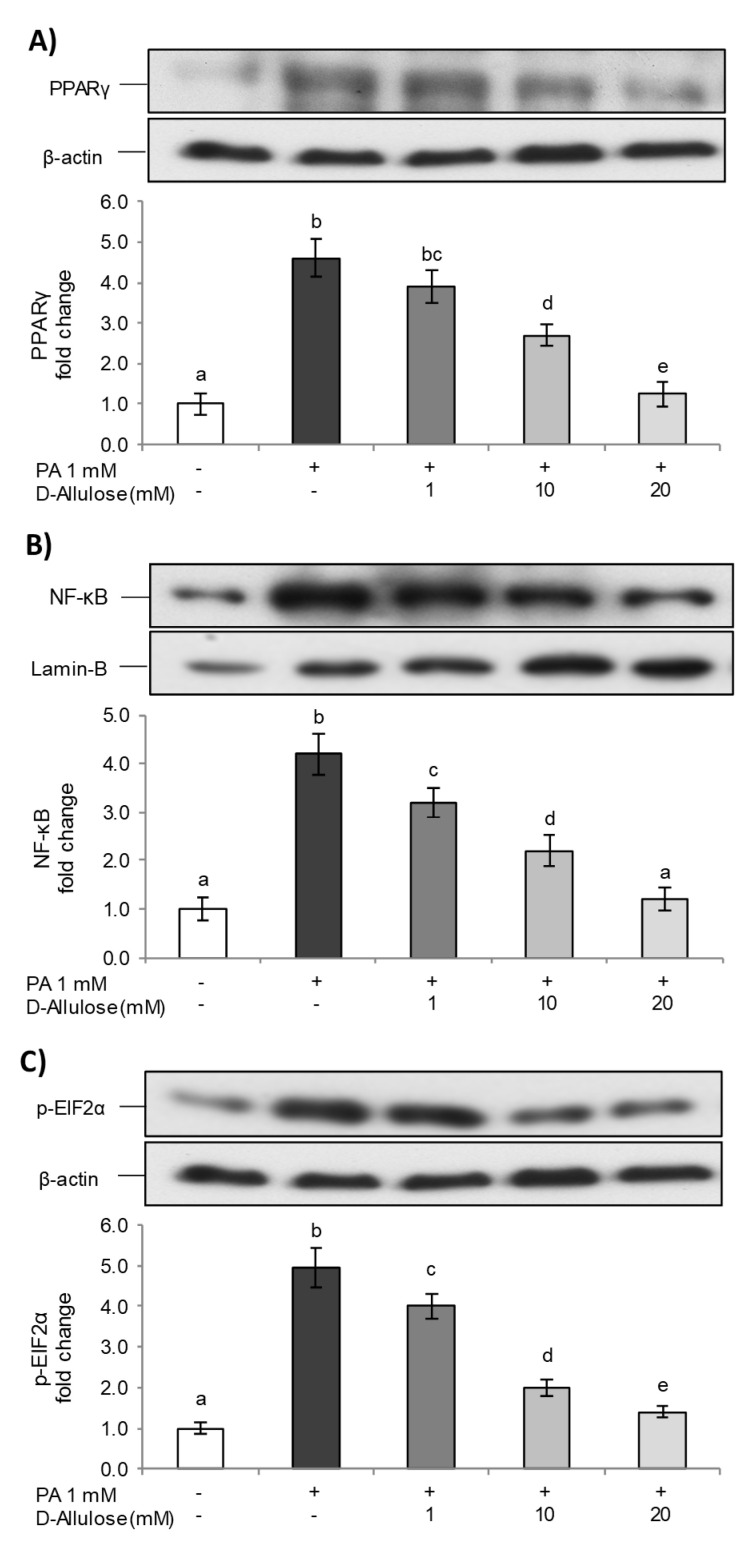
D-allulose ameliorates PA-induced adipocyte dysfunction. Totally differentiated 3T3-L1 cells were exposed for 24 h to 1 mM PA and then treated with medium containing D-allulose (1–10–20 mM) for further 24 h. Cells treated with the PA vehicle alone were used as controls. PPARγ (**A**), Nuclear NF-κB (**B**), and p-EIF2α (**C**) protein levels—the densitometry results are reported as fold change compared to controls. The intensity values of PPARγ, NF-κB, and p-EIF2α proteins were normalized to the corresponding value of β-actin or Lamin-B. All data are expressed as mean ± SD of three independent experiments (*n* = 3), each performed in triplicate. Means with the same letter are not significantly different from each other (*p* > 0.05).

As demonstrated above, excessive FFA levels are involved in the development of a lipotoxicity state that contributes to a chronic low-grade inflammatory condition and ER stress, thereby providing a direct link between obesity and several metabolic complications [32]. In order to further investigate D-allulose’s effects against obesity and related disorders, additional experiments were carried out, adding D-allulose in post treatment. Therefore, the cells were exposed for 24 h to 1 mM PA and then treated with a medium containing D-allulose (1–10–20 mM) for a further 24 h. In this step, the expression levels of PPARγ, NF-κB, and p-EIF2α were examined as representative markers of adipogenesis, inflammation, and ER stress. The results (Figure 6) show that the post treatment with D-allulose, at all tested concentrations and in a dose-dependent manner, was able to reduce the effects of PA, with a consequent reduction in the adipogenesis marker PPARγ, on the NF-κB inflammatory pathway, and, finally, the p-EIF2α levels, with a consequent inhibition of the UPR response and the restoration of ER homeostasis. Therefore, D-allulose was also able to protect adipocytes after PA-induced lipotoxicity, in the same way as was demonstrated for pretreatment conditions.

## 3. Discussion

Many studies have shown that a low-calorie diet has health benefits, improving weight loss, reducing cholesterol and triglycerides levels, preventing coronary heart disease, and slowing diabetes progression [33]. In this regard, there has been a high interest in natural sweeteners such as D-allulose, and in vitro and in vivo studies have demonstrated its metabolic effects as a regulator of glucose and fat metabolism, with antihyperglycemic, antihyperlipidemic, and anti-inflammatory properties and consequent effects against obesity and its pathological consequences [34]. Furthermore, D-allulose reduced weight and BMI and weight change in overweight or obese patients with T2DM after 8 weeks of supplementation [35]. 

Our results demonstrate that treatment with D-allulose in substitution of glucose was able to prevent, at all the concentrations tested, adipocyte hypertrophy induced by high concentrations of PA (Figure 1). In fact, it reduced lipid accumulation in adipocytes exposed to PA in a dose-dependent way. Interestingly, we confirmed that D-allulose effects were associated with reduced C/EBP-β and PPARγ expression, important adipogenic factors [34]. To the same extent, we further demonstrated that D-allulose reduced PA-induced *FABP4*, *SREBP1*, and *FASN* gene expression, which plays a key role in fatty acid transportation and metabolism, and lipid storage in adipose tissue [36]. D-allulose’s effects on adipocyte dysfunction have been demonstrated in other dysfunctional disorders or treatment conditions. Chen and coworkers reported that the concomitant supplementation of D-allulose and high carbohydrate intake inhibited fat accumulation in rats with a minimum body weight increase, less epididymal fat, and smaller adipose cell size [37]. Similarly, D-allulose reduced C/EBP-β and PPARγ expression during the adipogenesis process in murine adipocytes [17]. 

In obesity, the abnormal expansion of adipose tissue is accompanied by inflammatory changes, contributing to the onset of a chronic low-grade systemic inflammation state [38]. Our data show that PA activated the NF-κB pathway via pIKK α/β and induced the transcription machinery of genes involved in inflammation, such as *IL-6* and *IL-8*. Inversely, in our experimental conditions, D-allulose pretreatment was able to inhibit the NF-κB inflammatory pathway induced by PA (Figure 3A,B). These effects were also confirmed at a transcriptional level, since D-allulose reduced, in a dose-dependent way, the gene expression of *IL-6* and *IL-8* (Figure 3C,D).

It is known that saturated long-chain fatty acids, such as PA, accumulated into adipocytes, are metabolized into lysophosphatidylcholine, diacylglycerol, and ceramides. In hypertrophic and dysfunctional adipocytes, high concentrations of these compounds induce PKC activation and ER stress [39,40]. In our experimental conditions, PA induced ROS levels [41], whereas D-allulose pretreatment prevented ROS production in adipocytes exposed to PA in a dose-dependent way. This effect is probably mediated by the ability of D-allulose to activate an antioxidant adaptive response via Nrf2/HO-1 pathway activation (Figure 4). Additionally, we confirmed that PA exposure produced ER stress, as reported elsewhere [41], inducing typical markers of this adaptive response, such as XBP-1s and p-EIF2a, as well as the mRNA levels of *GRP-78* and *CHOP*, which initiate the UPR to restore cellular homeostasis. Also, in this case, D-allulose pretreatment reduced, in a dose-dependent way, ER stress markers, restoring their levels to those of the control at the higher concentration tested. These data have also been confirmed in other experimental models, since naturally occurring rare sugars have been demonstrated to bear free radical scavenger activities and to reduce ER stress [42].

In our study, we additionally performed a set of experiments involving D-allulose post treatment in order to evaluate its efficacy not only in a preventive approach but also in a therapeutic one. With this aim, we selected representative markers of adipogenesis, inflammation, and ER stress to compare D-allulose effects during the two different treatment periods. D-Allulose treatment after PA exposure exerted the same effects of pretreatment, since it dose-dependently reduced the levels of the main adipogenesis marker PPARγ. Additionally, D-allulose post treatment was also effective in reducing NF-κB nuclear translocation and ER stress activation at all the concentrations tested. Also, in vivo studies in genetically obese C57BL/6J-ob/ob mice demonstrated that D-allulose treatment alters the networks of inflammatory response and lipid metabolism [43] via the modulation of 103 differentially expressed genes involved in the inflammatory response, molecular transport, and lipid metabolism [43].

One aspect of our research that deserves discussion is the choice of D-allulose concentration range used in our experiments. Daniel et al. [44] hypothesize that a mean intake of D-allulose of about 400 mg/kg·bw per day from enriched products could be expected for adult individuals. The literature reports the results of clinical studies conducted using very different doses of D-allulose, even up to 15 g per day. For example, in the paper by Fukunaga et al. [45], a diabetic diet containing 8.5 g D-allulose was found to improve postprandial glucose levels in type two diabetics compared with a normal diabetic diet. A recent metanalysis by Yuma et al. [46] reported a comparison between a D-allulose intake group and a control group, showing that both the 5 g and 10 g intake groups had a significantly smaller area under the curve of postprandial blood glucose levels. Franchi et al. [47] assessed the effects of D-allulose in non-diabetic Westerners given D-allulose (2.5, 5.0, 7.5, 10.0 g), demonstrating an early dose-dependent reduction in plasma glucose and insulin levels, as well as decreased postprandial glucose and insulin excursion. Finally, in a small clinical study in people with impaired glucose metabolism, an increase in total and LDL-cholesterol was observed after a 12-week intake of 15 g D-allulose per day [48]. However, regarding the development of side effects following D-allulose ingestion, Han et al. [19] reported the results of a non-randomized controlled trial carried out on healthy and young subjects and showed that increasing the total daily D-allulose intake gradually to 1.0 g/kg·bw for regular ingestion resulted in incidences of severe nausea, abdominal pain, headache, anorexia, and diarrheal symptoms. Based on these results, the authors suggested that a maximum single dose and maximum total daily intake of D-allulose of 0.4 g/kg·bw and 0.9 g/kg·bw, respectively, may be taken into consideration. In our study, we used concentrations ranging between 1 and 20 mM. In all experiments, the beneficial effect of D-allulose against PA-induced lipotoxicity was statistically evident already at a dose as low as 1 mM, mimicking a plasmatic concentration that may be obtained through the consumption of allulose-based foods or allulose dietary supplementation. On the other hand, the higher dose employed in our study (20 mM) seems to remain below the potential plasma concentrations of D-allulose capable of inducing gastrointestinal side effects, and, at the same time, allows us to more clearly highlight the molecular mechanisms that underlie the protective effects of D-allulose against adipocyte dysfunction.

## 4. Materials and Methods

### 4.1. Reagents

Dimethyl sulfoxide (DMSO), 2-propanol, ethanol, and methanol, in their highest commercially available purity grade, were purchased from VWR (Milan, Italy). D-allulose (molecular formula, C_6_H_12_O_6_), palmitic acid (PA), bovine serum albumin (BSA), fetal bovine serum (FBS), Dulbecco′s Modified Eagle′s Medium without glucose, 3-iso-butyl-1 methylxanthine (IBMX), dexamethasone (DEX), and all the other chemicals, unless otherwise specified, were purchased from Merck life Science (Milan, Italy). 

Lipid-containing medium was prepared via conjugation of PA to BSA using a modified method set in our laboratory [49]. Briefly, PA was first dissolved in ethanol at 200 mmol/L and then combined with 10% FFA-free BSA to a 4 mM final concentration at 60 °C before use. Then, the mixture was gently agitated to dissolve the PA-albumin complexes, pH was adjusted to 7.4 with 1 N NaOH, and, finally, the solution was filter-sterilized. Final ethanol concentration was 0.05% *w*/*w*.

### 4.2. Cell Culture and Treatments

The 3T3-L1 murine preadipocytes were obtained from the American Type Culture Collection (Manassas, VA, USA) and cultured in Dulbecco’s Modified Essential Medium (DMEM) supplemented with 10% newborn calf serum, 4 mm L-glutamine, 100-unit/mL penicillin/streptomycin and 25 mm HEPES buffer. The cells were maintained at 37 °C in a humidified atmosphere with 95% air and 5% CO_2_. 

To achieve mature adipocytes, 3T3-L1 cells were seeded in DMEM growth medium, supplemented with 10% FBS, 4 mM L-glutamine, 100 U/mL penicillin/streptomycin solution, and 25 mM HEPES buffer, at 1.3 × 10^4^ cells/cm^2^ in multiwell plate and cultured for 10 days after confluence.

In detail, during the differentiation process, the cells were treated for the first 4 days in DMEM growth medium containing prodifferentiation agents (0.5 mM IBMX, 1 μΜ DEX, and 1 μg/mL insulin); subsequently, from day 4 to day 7, we added a medium containing only 1 μg/mL insulin, while from day 7 to day 10, the cells were maintained in an insulin-free DMEM up the total differentiation in mature adipocytes. In the experiments, cells were always used within the 20th passage.

To evaluate the protective effect of D-allulose against a lipotoxicity state induced in adipose tissue, fully differentiated 3T3-L1 adipocytes were subjected to pretreatment for 24 h with a medium containing D-allulose at three different concentrations (1–10–20 mM), prepared by replacing the total amount of glucose in the DMEM (25 mM) with the corresponding concentration of D-allulose. The medium containing D-allulose was always freshly prepared and immediately used. After this incubation time, 3T3-L1 cells were subject to rapid washing with Dulbecco’s phosphate-buffered solution (DPBS) and then, in order to investigate lipotoxicity, were either exposed or not for 24 h to PA 1 mM.

Moreover, in order to investigate D-allulose’s effect towards a lipotoxicity state already induced in adipose tissue, the totally differentiated 3T3-L1 cells were firstly exposed for 24 h to 1 mM PA and then treated, for further 24 h, with the medium containing D-allulose (1–10–20 mM), prepared as previously described.

For all experiments, cells treated with DMEM (containing 25 mM of glucose) and exposed only to the PA vehicle were used as controls. 

D-Allulose concentrations used in our experiments (1–10–20 mM) were selected according to previous in vitro studies on adipocytes [16]; these D-allulose amounts, added to the medium in substitution of the same amounts of glucose and tested on 3T3-L1 adipocytes not exposed to PA under the same experimental conditions, produced no change, in comparison with controls, in cellular lipid accumulation and in the markers of adipogenesis, inflammation, oxidative stress, and ER stress. Furthermore, no significant metabolic changes were found in 3T3-L1 cells when cultured in a medium containing different concentrations of glucose (5–15–25 mM), simulating the glucose amounts present in D-allulose treatments (see Supplemental Materials).

### 4.3. Oil Red O Staining

D-allulose’s effect on adipogenesis was assessed via Oil Red O histological staining technique according to the method described by Molonia and coworkers [50]. After staining, the cells were photographed (40× magnification) and the extent of lipid accumulation was determined by eluting the Oil Red O stain retained in the cells with 100% isopropanol. The lipid accumulation was finally quantified via spectrophotometric analysis at 490 nm, and results were expressed as fold change against control.

### 4.4. Cell Lysate Preparation

After the appropriate treatments, 3T3-L1 cells were rinsed with DPBS and harvested with a scraper. Total, nuclear, or cytoplasmic proteins were extracted, as previously described [51]. Briefly, to obtain nuclear and cytosolic extracts, cells were incubated for 15 min at 4 °C in a hypotonic buffer (10 mM HEPES, pH 7.9, 10 mM KCl, 1.5 mM MgCl_2_, and 5% glycerol) supplemented with protease inhibitors (1 μg/mL leupeptine, 2 μg/mL aprotinine, 1 mM benzamidine, and 5 mM NaF) and 1 mM dithiothreitol (DTT) and treated with 0.60% Igepal. Following centrifugation at 13,000 rpm for 1 min at 4 °C, the supernatant was collected as the cytoplasmic extract, while nuclear proteins were extracted from the pellet using a hypertonic buffer (20 mM HEPES, 400 mM NaCl, 1 mM MgCl_2_, 1 mM EGTA, 0.1 mM EDTA, and 10% glycerol) containing the same protease inhibitors and DTT. Alternatively, total lysate was prepared by treating harvested cells with a lysis buffer (10 mM Tris–HCl, 1 mM EDTANa2, 150 mM NaCl, 5% glycerol, 0.1% SDS, and 1% Tryton) supplemented with protease inhibitors (1 μg/mL leupeptine, 2 μg/mL aprotinine, 1 mM benzamidine, and 5 mM NaF) and 1 mM DTT. The protein fractions were stored at −20 °C until further use, and the protein concentration in lysates was assessed using the Bradford assay [52].

### 4.5. Immunoblotting

For immunoblot analyses, the proteic lysates were denatured in 4× SDS-PAGE sample buffer (260 mM Tris–HCl, pH 8.0, 40% (*v*/*v*) glycerol, 9.2% (*w*/*v*) SDS, 0.04% bromo-phenol blue, and 2-mercaptoethanol as reducing agent) and subjected to SDS-PAGE on 10–12% acrylamide/ bisacrylamide gels. After separation the proteins were transferred to Polyvinylidene Difluoride (PVDF) membranes (Hybond-P PVDF, Amersham Bioscience, Milan, Italy). Membranes were then incubated overnight with specific primary antibodies: mouse anti-PPAR-γ monoclonal antibody (Santa Cruz Biotechnology, Santa Cruz, CA, USA) (1:1500), rabbit anti- C/EBPβ (LAP) polyclonal antibody (Cell Signaling Technology, Danvers, MA, USA) (1:1000), rabbit anti-NF-κB p65 polyclonal antibody (Invitrogen, Waltham, MA,USA) (1:1000), rabbit anti-Phospho-IKK α/β (Ser176/180) monoclonal antibody (Cell Signaling Technology, Danvers, MA, USA) (1:1000), rabbit anti-Nrf2 polyclonal antibody (Santa Cruz Biotechnology, Santa Cruz, CA, USA) (1:1000), rabbit anti-XBP-1s (E9V3E) monoclonal antibody (Cell Signaling Technology, Danvers, MA, USA) (1:500), rabbit anti-Phospho-EIF2α (Ser51) (D9G8) monoclonal antibody (Cell Signaling Technology, Danvers, MA, USA) (1:500), rabbit anti-β-Actin monoclonal antibody (Cell Signaling Technology, Danvers, MA, USA) (1:6000), rabbit anti-Lamin-B monoclonal antibody (Cell Signaling Technology, Danvers, MA, USA) (1:1500), followed by 2 h incubation with peroxidase-conjugated secondary antibody HRP labeled goat antirabbit Ig (Cell Signaling Technology, Danvers, MA, USA) (1:6000), goat anti-Mouse IgM Secondary Antibody, HRP conjugate (Cell Signaling Technology, Danvers, MA, USA) (1:6000) and visualized with an ECL plus detection system (Amersham Biosciences, Milan, Italy). The protein bands were detected using high-performance chemiluminescence film (Amersham Hyperfilm™ ECL; GE Healthcare Life Sciences, Little Chalfont, UK). Quantitative analysis was performed via densitometry. The equivalent loading of proteins in each well was confirmed via Ponceau staining and β-actin or Lamin B control.

### 4.6. Real-Time PCR

Total cellular RNA was extracted using E.Z.N.A.^®^ Total RNA kit according to manufacturer’s instruction (OMEGA Bio-Tek, VWR,Norcross, GA, USA), quantified via Quanti-iT TM RNA assay kit QUBIT (Invitrogen, Milan, Italy), and reverse-transcribed with M-MLV Reverse Transcriptase. Quantitative real-time polymerase chain reaction (PCR; Applied Biosystems 7300 Real-Time PCR System, CA, USA) coupled with SYBR green chemistry (SYBR green JumpStart Taq Ready Mix) was performed for identification of mRNA levels of *FABP4* (FW 5′-AAG GTG AAG AGC ATC ATA ACC CT-3′, RV 5′-TCA CGC CTT TCA TAA CAC ATT CC-3′) [53], *SREBP-1* (FW 5′-TGG CTT GGT GAT GCT ATG TT -3′, RV 5′- TAA GGG GTT GGG AGT AGA GG -3′) [54], *FASN* (FW 5′-GGA GGT GGT GAT AGC CGG TAT-3′, RV 5′- TGG GTA ATC CAT AGA GCC CAG-3′) [55], *IL-6* (FW 5′-GAT GGA TGC TAC CAA ACT GGA T-3′, RV 5′-CCA GGT AGC TAT GGT ACT CCA GA-3′) [53], *IL-8* (FW 5′- GCA CTT GGG AAG TTA ACG CA-3′, RV 5′- GCA CAG TGT CCC TAT AGC CC-3′) [51], *HO-1* (FW 5′-ACA TCG ACA GCC CCA CCA AGT TCA A-3′, RV 5′-CTG ACG AAG TGA CG CCA TCT GTG AG-3′) [56], *CHOP* (FW 5′-CGGAACCTGAGGAGAGAGTG-3’, RV 5′-TATAGGTGCCCCCAATTTCA-3′) [57], *GRP78* (FW 5′-GGATGCGGACATTGAAGACT-3’, RV 5′-TCCCAACGAAAGTTCCTGAG-3’) [58]. *18S* rRNA (FW 5′-GTAACCCGTTGAACCCCATT-3′, RV 5′-CCATCCAATCGGTAGTAGCG-3′) [59] was used as reference gene. Data were elaborated by SDS 1.3.1 software (Applied Biosystems, Foster City, CA, USA) and expressed as threshold cycle (Ct). The fold increase in mRNA expression compared with control cells was determined using the 2^−ΔΔCt^ method [60].

### 4.7. ROS Measurement via Dichlorodihydro-Fluorescein Diacetate Assay

The intracellular reactive oxygen species (ROS) levels were measured using dichloro-dihydro-fluorescein diacetate (DCFH-DA) according to the method previously described [61]. Briefly, following treatment, the cells were washed twice with DPBS and incubated, in the dark, with DCFH-DA 50 μM at 37 °C for 30 min. The lipophilic non-fluorescent DCFH-DA readily crosses the cell membrane through passive diffusion followed by deacetylation. The deacylated product is the non-fluorescent oxidant-sensitive DCHF, which is oxidized later by intracellular ROS to form highly fluorescent DCF, which is measured. The intensity of fluorescence was detected at an excitation wavelength of 485 nm and an emission wavelength of 530 nm using a microplate reader (GloMax^®^ Discover System-TM397, Madison, WI, USA). The fluorescence intensity is directly proportional to the ROS amount. ROS levels were expressed as DCF relative fluorescence intensity per mg of proteins against control.

### 4.8. Statistical Analysis

All the experiments were performed in triplicate and repeated three times. Results are expressed as mean ± SD from three experiments and statistically analyzed via one-way ANOVA test, followed by Tukey’s HSD, using the statistical software ezANOVA version 0.985 (https://people.cas.sc.edu/rorden/ezanova/index.html (accessed on 6 November 2023)). Differences in groups and treatments were considered significant for *p* < 0.05.

## 5. Conclusions

Although we are aware that the adipose tissue is a complex and heterogeneous organ, with many cell types and circulating factors interacting through different signaling pathways, the present findings suggest that D-allulose, when used as substitute for glucose in the diet, might have an important protective role in adipocyte functions and help to clarify the molecular mechanism of action of this sugar as a preventive or therapeutic compound against PA lipotoxicity through the modulation of specific cellular pathways connected to lipid transport and metabolism systems. However, we recognize that these data may not fully reflect the effects of D-allulose in our organism; therefore, further studies will be necessary to support these data in co-culture systems and/or in vivo.

## Data Availability

The data that support the findings of this study are available on reasonable request from the corresponding author (Antonio Speciale).

## Supplementary Materials

The following supporting information can be downloaded at: https://www.mdpi.com/article/10.3390/ijms25074059/s1.

## References

[1] Longo M., Zatterale F., Naderi J., Parrillo L., Formisano P., Raciti G.A., Beguinot F., Miele C. (2019). Adipose Tissue Dysfunction as Determinant of Obesity-Associated Metabolic Complications. Int. J. Mol. Sci..

[2] Ebbert J.O., Jensen M.D. (2013). Fat depots, free fatty acids, and dyslipidemia. Nutrients.

[3] Stenkula K.G., Erlanson-Albertsson C. (2018). Adipose cell size: Importance in health and disease. Am. J. Physiol. Regul. Integr. Comp. Physiol..

[4] Piché M.E., Tchernof A., Després J.P. (2020). Obesity Phenotypes, Diabetes, and Cardiovascular Diseases. Circ. Res..

[5] Khanna D., Khanna S., Khanna P., Kahar P., Patel B.M. (2022). Obesity: A Chronic Low-Grade Inflammation and Its Markers. Cureus.

[6] Ertunc M.E., Hotamisligil G.S. (2016). Lipid signaling and lipotoxicity in metaflammation: Indications for metabolic disease pathogenesis and treatment. J. Lipid Res..

[7] Jacquemyn J., Cascalho A., Goodchild R.E. (2017). The ins and outs of endoplasmic reticulum-controlled lipid biosynthesis. EMBO Rep..

[8] Mooradian A.D., Smith M., Tokuda M. (2017). The role of artificial and natural sweeteners in reducing the consumption of table sugar: A narrative review. Clin. Nutr. ESPEN.

[9] Mortensen A. (2006). Sweeteners permitted in the European Union: Safety aspects. Scand. J. Food Nutr..

[10] Smith A., Avery A., Ford R., Yang Q., Goux A., Mukherjee I., Neville D.C.A., Jethwa P. (2021). Rare sugars: Metabolic impacts and mechanisms of action: A scoping review. Br. J. Nutr..

[11] Ahmed A., Khan T.A., Dan Ramdath D., Kendall C.W.C., Sievenpiper J.L. (2022). Rare sugars and their health effects in humans: A systematic review and narrative synthesis of the evidence from human trials. Nutr. Rev..

[12] Whistler R.L., Singh P.P., Lake W.C. (1974). D-Psicose metabolism in the rat. Carbohydr. Res..

[13] Iida T., Hayashi N., Yamada T., Yoshikawa Y., Miyazato S., Kishimoto Y., Okuma K., Tokuda M., Izumori K. (2010). Failure of d-psicose absorbed in the small intestine to metabolize into energy and its low large intestinal fermentability in humans. Metabolism.

[14] Maeng H.J., Yoon J.H., Chun K.H., Kim S.T., Jang D.J., Park J.E., Kim Y.H., Kim S.B., Kim Y.C. (2019). Metabolic Stability of D-Allulose in Biorelevant Media and Hepatocytes: Comparison with Fructose and Erythritol. Foods.

[15] Iwasaki Y., Sendo M., Dezaki K., Hira T., Sato T., Nakata M., Goswami C., Aoki R., Arai T., Kumari P. (2018). GLP-1 release and vagal afferent activation mediate the beneficial metabolic and chronotherapeutic effects of D-allulose. Nat. Commun..

[16] Lee G.H., Peng C., Lee H.Y., Park S.A., Hoang T.H., Kim J.H., Sa S., Kim G.E., Han J.S., Chae H.J. (2021). D-allulose ameliorates adiposity through the AMPK-SIRT1-PGC-1α pathway in HFD-induced SD rats. Food Nutr. Res..

[17] Moon S., Kim Y.H., Choi K. (2020). Inhibition of 3T3-L1 Adipocyte Differentiation by D-allulose. Biotechnol. Bioprocess Eng..

[18] Ambele M.A., Dhanraj P., Giles R., Pepper M.S. (2020). Adipogenesis: A Complex Interplay of Multiple Molecular Determinants and Pathways. Int. J. Mol. Sci..

[19] Han Y., Choi B.R., Kim S.Y., Kim S.B., Kim Y.H., Kwon E.Y., Choi M.S. (2018). Gastrointestinal Tolerance of D-Allulose in Healthy and Young Adults. A Non-Randomized Controlled Trial. Nutrients.

[20] Furuhashi M., Saitoh S., Shimamoto K., Miura T. (2014). Fatty Acid-Binding Protein 4 (FABP4): Pathophysiological Insights and Potent Clinical Biomarker of Metabolic and Cardiovascular Diseases. Clin. Med. Insights Cardiol..

[21] Kawai T., Autieri M.V., Scalia R. (2021). Adipose tissue inflammation and metabolic dysfunction in obesity. Am. J. Physiol. Cell Physiol..

[22] Baker R.G., Hayden M.S., Ghosh S. (2011). NF-κB, inflammation, and metabolic disease. Cell Metab..

[23] Han M.S., White A., Perry R.J., Camporez J.P., Hidalgo J., Shulman G.I., Davis R.J. (2020). Regulation of adipose tissue inflammation by interleukin 6. Proc. Natl. Acad. Sci. USA.

[24] Marseglia L., Manti S., D’Angelo G., Nicotera A., Parisi E., Di Rosa G., Gitto E., Arrigo T. (2014). Oxidative stress in obesity: A critical component in human diseases. Int. J. Mol. Sci..

[25] Sankhla M., Sharma T.K., Mathur K., Rathor J.S., Butolia V., Gadhok A.K., Vardey S.K., Sinha M., Kaushik G.G. (2012). Relationship of oxidative stress with obesity and its role in obesity induced metabolic syndrome. Clin. Lab..

[26] Naomi R., Teoh S.H., Embong H., Balan S.S., Othman F., Bahari H., Yazid M.D. (2023). The Role of Oxidative Stress and Inflammation in Obesity and Its Impact on Cognitive Impairments—A Narrative Review. Antioxidants.

[27] Saha S., Buttari B., Panieri E., Profumo E., Saso L. (2020). An Overview of Nrf2 Signaling Pathway and Its Role in Inflammation. Molecules.

[28] Ahmed S.M., Luo L., Namani A., Wang X.J., Tang X. (2017). Nrf2 signaling pathway: Pivotal roles in inflammation. Biochim. Biophys. Acta Mol. Basis Dis..

[29] Celik C., Lee S.Y.T., Yap W.S., Thibault G. (2023). Endoplasmic reticulum stress and lipids in health and diseases. Prog. Lipid Res..

[30] Han J., Back S.H., Hur J., Lin Y.H., Gildersleeve R., Shan J., Yuan C.L., Krokowski D., Wang S., Hatzoglou M. (2013). ER-stress-induced transcriptional regulation increases protein synthesis leading to cell death. Nat. Cell Biol..

[31] Ibrahim I.M., Abdelmalek D.H., Elfiky A.A. (2019). GRP78: A cell’s response to stress. Life Sci..

[32] Jung U.J., Choi M.S. (2014). Obesity and its metabolic complications: The role of adipokines and the relationship between obesity, inflammation, insulin resistance, dyslipidemia and nonalcoholic fatty liver disease. Int. J. Mol. Sci..

[33] Feingold K.R. (2021). The bidirectional link between HDL and COVID-19 infections. J. Lipid Res..

[34] Chen Z., Gao X.D., Li Z. (2022). Recent Advances Regarding the Physiological Functions and Biosynthesis of D-Allulose. Front. Microbiol..

[35] Tak J., Bok M., Rho H., Park J.H., Lim Y., Chon S., Lim H. (2023). Effect of diabetes-specific oral nutritional supplements with allulose on weight and glycemic profiles in overweight or obese type 2 diabetic patients. Nutr. Res. Pract..

[36] Guo L., Li X., Tang Q.Q. (2015). Transcriptional regulation of adipocyte differentiation: A central role for CCAAT/enhancer-binding protein (C/EBP) β. J. Biol. Chem..

[37] Chen J., Huang W., Zhang T., Lu M., Jiang B. (2019). Anti-obesity potential of rare sugar d-psicose by regulating lipid metabolism in rats. Food Funct..

[38] Chait A., den Hartigh L.J. (2020). Adipose Tissue Distribution, Inflammation and Its Metabolic Consequences, Including Diabetes and Cardiovascular Disease. Front. Cardiovasc. Med..

[39] Cinti S. (2023). Obese Adipocytes Have Altered Redox Homeostasis with Metabolic Consequences. Antioxidants.

[40] Ajoolabady A., Lebeaupin C., Wu N.N., Kaufman R.J., Ren J. (2023). ER stress and inflammation crosstalk in obesity. Med. Res. Rev..

[41] Gao C.L., Zhu C., Zhao Y.P., Chen X.H., Ji C.B., Zhang C.M., Zhu J.G., Xia Z.K., Tong M.L., Guo X.R. (2010). Mitochondrial dysfunction is induced by high levels of glucose and free fatty acids in 3T3-L1 adipocytes. Mol. Cell. Endocrinol..

[42] Mooradian A.D., Haas M.J., Onstead-Haas L., Tani Y., Iida T., Tokuda M. (2020). Naturally occurring rare sugars are free radical scavengers and can ameliorate endoplasmic reticulum stress. Int. J. Vitam. Nutr. Res..

[43] Kim S.-E., Kim S.J., Kim H.-J., Sung M.-K. (2017). d-Psicose, a sugar substitute, suppresses body fat deposition by altering networks of inflammatory response and lipid metabolism in C57BL/6J-ob/ob mice. J. Funct. Foods.

[44] Daniel H., Hauner H., Hornef M., Clavel T. (2022). Allulose in human diet: The knowns and the unknowns. Br. J. Nutr..

[45] Fukunaga K., Yoshimura T., Imachi H., Kobayashi T., Saheki T., Sato S., Saheki N., Jiang W., Murao K. (2023). A Pilot Study on the Efficacy of a Diabetic Diet Containing the Rare Sugar D-Allulose in Patients with Type 2 Diabetes Mellitus: A Prospective, Randomized, Single-Blind, Crossover Study. Nutrients.

[46] Yuma T., Tokuda M., Nishimoto N., Yokoi H., Izumori K. (2023). Allulose for the attenuation of postprandial blood glucose levels in healthy humans: A systematic review and meta-analysis. PLoS ONE.

[47] Franchi F., Yaranov D.M., Rollini F., Rivas A., Rivas Rios J., Been L., Tani Y., Tokuda M., Iida T., Hayashi N. (2021). Effects of D-allulose on glucose tolerance and insulin response to a standard oral sucrose load: Results of a prospective, randomized, crossover study. BMJ Open Diabetes Res. Care.

[48] Tanaka M., Kanasaki A., Hayashi N., Iida T., Murao K. (2020). Safety and efficacy of a 48-week long-term ingestion of D-allulose in subjects with high LDL cholesterol levels. Fundam. Toxicol. Sci..

[49] Molonia M.S., Occhiuto C., Muscarà C., Speciale A., Ruberto G., Siracusa L., Cristani M., Saija A., Cimino F. (2022). Effects of a pinitol-rich Glycyrrhiza glabra L. leaf extract on insulin and inflammatory signaling pathways in palmitate-induced hypertrophic adipocytes. Nat. Prod. Res..

[50] Molonia M.S., Speciale A., Muscarà C., Salamone F.L., Saija A., Cimino F. (2024). Low concentrations of α-lipoic acid reduce palmitic acid-induced alterations in murine hypertrophic adipocytes. Nat. Prod. Res..

[51] Molonia M.S., Muscarà C., Speciale A., Salamone F.L., Costa G., Vento G., Saija A., Cimino F. (2023). Low concentrations of antimony impair adipogenesis and endoplasmic reticulum homeostasis during 3T3-L1 cells differentiation. Food Chem. Toxicol..

[52] Bradford M.M. (1976). A rapid and sensitive method for the quantitation of microgram quantities of protein utilizing the principle of protein-dye binding. Anal. Biochem..

[53] Molonia M.S., Muscarà C., Speciale A., Salamone F.L., Toscano G., Saija A., Cimino F. (2022). The p-Phthalates Terephthalic Acid and Dimethyl Terephthalate Used in the Manufacture of PET Induce In Vitro Adipocytes Dysfunction by Altering Adipogenesis and Thermogenesis Mechanisms. Molecules.

[54] Ning C., Li G., You L., Ma Y., Jin L., Ma J., Li X., Li M., Liu H. (2017). MiR-185 inhibits 3T3-L1 cell differentiation by targeting SREBP-1. Biosci. Biotechnol. Biochem..

[55] Zhang Y., Yu H., Gao P., Chen J., Yu C., Zong C., Lu S., Li X., Ma X., Liu Y. (2016). The Effect of Growth Hormone on Lipid Accumulation or Maturation in Adipocytes. Cell. Physiol. Biochem..

[56] Sun J., Brand M., Zenke Y., Tashiro S., Groudine M., Igarashi K. (2004). Heme regulates the dynamic exchange of Bach1 and NF-E2-related factors in the Maf transcription factor network. Proc. Natl. Acad. Sci. USA.

[57] Kavalakatt S., Khadir A., Madhu D., Koistinen H.A., Al-Mulla F., Tuomilehto J., Abubaker J., Tiss A. (2021). Urocortin 3 overexpression reduces ER stress and heat shock response in 3T3-L1 adipocytes. Sci. Rep..

[58] Ryu K.Y., Jeon E.J., Leem J., Park J.H., Cho H. (2020). Regulation of Adipsin Expression by Endoplasmic Reticulum Stress in Adipocytes. Biomolecules.

[59] Molonia M.S., Quesada-Lopez T., Speciale A., Muscarà C., Saija A., Villarroya F., Cimino F. (2021). In Vitro Effects of Cyanidin-3-O-Glucoside on Inflammatory and Insulin-Sensitizing Genes in Human Adipocytes Exposed to Palmitic Acid. Chem. Biodivers..

[60] Livak K.J., Schmittgen T.D. (2001). Analysis of relative gene expression data using real-time quantitative PCR and the 2(-Delta Delta C(T)) Method. Methods.

[61] Bashllari R., Molonia M.S., Muscarà C., Speciale A., Wilde P.J., Saija A., Cimino F. (2023). Cyanidin-3-O-glucoside protects intestinal epithelial cells from palmitate-induced lipotoxicity. Arch. Physiol. Biochem..

