# Supplementary material for: D-Allulose Reduces Hypertrophy and Endoplasmic Reticulum Stress Induced by Palmitic Acid in Murine 3T3-L1 Adipocytes
Source: Int J Mol Sci. 2024 Apr 5;25(7):4059. doi: 10.3390/ijms25074059 (PMC11012259; doi:10.3390/ijms25074059)
Supplement: Supplementary file 1 [file ijms-25-04059-s001.zip › Supplementary Material 1.pdf]

Supplementary material

D-Allulose Reduces Hypertrophy and Endoplasmic Reticulum Stress Induced by Palmitic Acid in Murine 3T3-L1 Adipocytes

Maria Sofia Molonia [1,2], Federica Lina Salamone [1], Antonio Speciale [1,*], Antonella Saija [1,†] and Francesco Cimino [1,†]

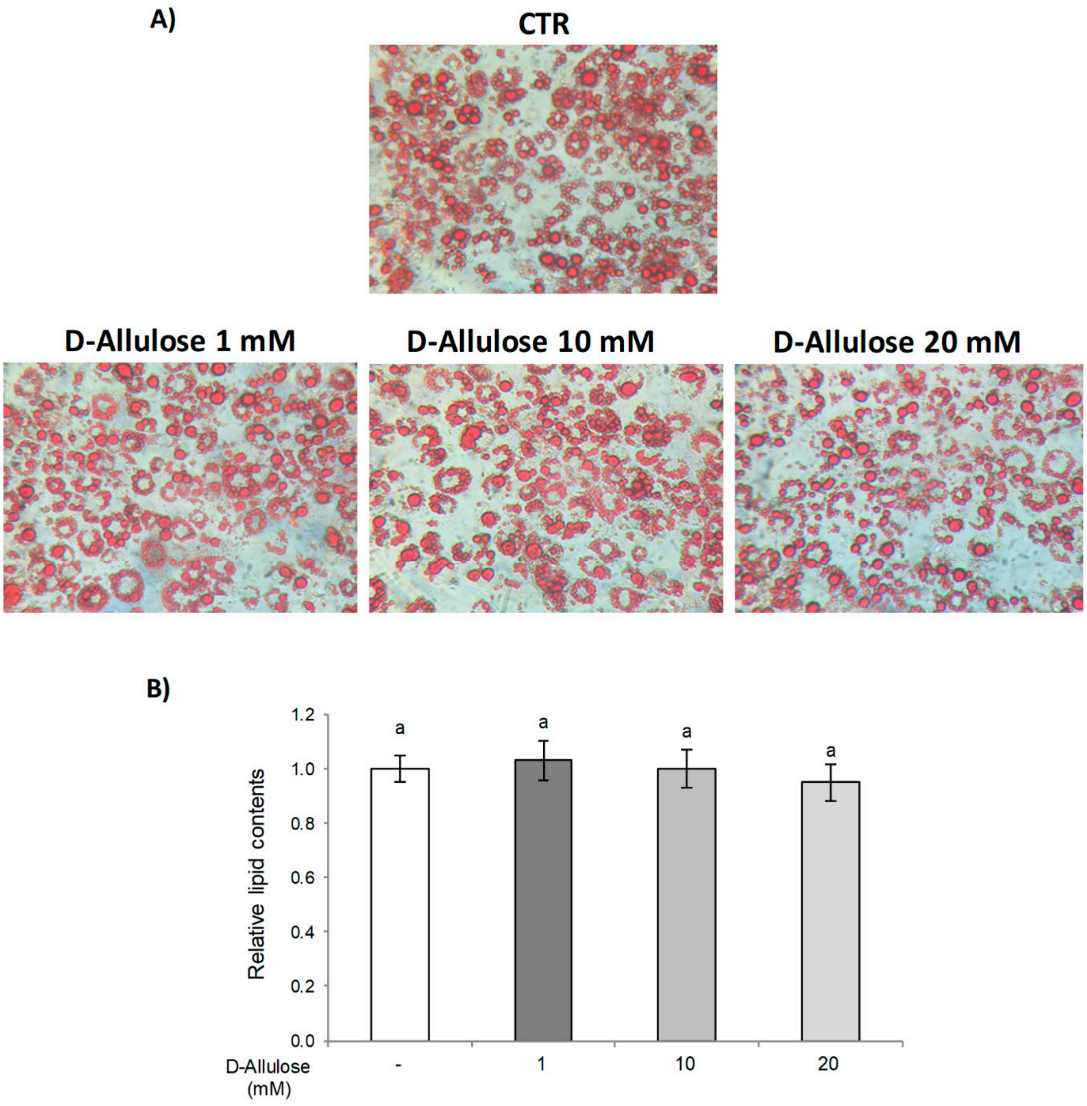

Figure S1. Totally differentiated 3T3-L1 cells were cultured for 24 h in a medium containing D-allulose (1-10-20 mM) and treated for 24 h only with the PA vehicle (see material and methods section for more details). Cells treated with the PA vehicle alone were used as controls. (A) Staining with Oil Red O (original magnification at 40X). Representative images of three independent experiments. (B) Quantitative assessment of Oil red O content. All data are expressed as mean ± SD of three independent experiments (n = 3) each performed in triplicate. Means with the same letter are not significantly different from each other ($p > 0.05$).

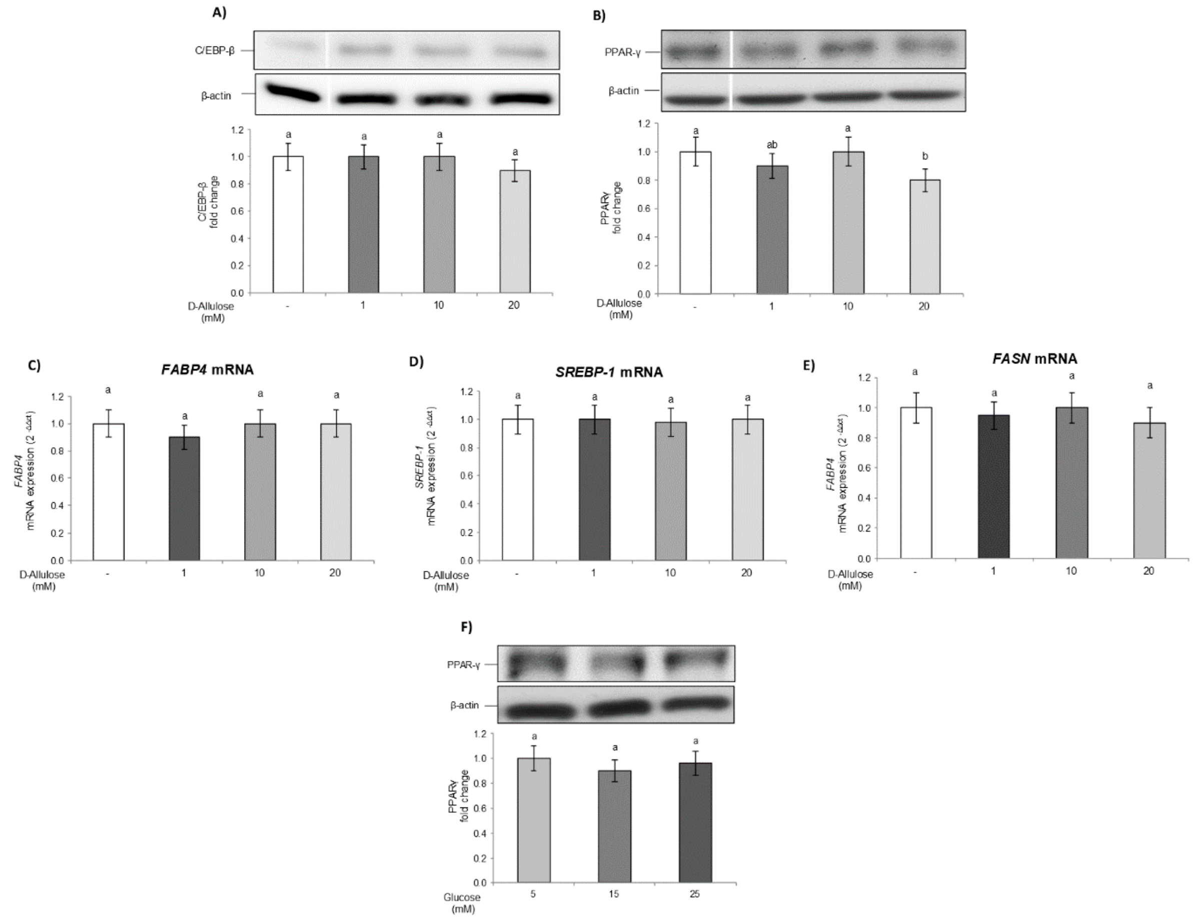

Figure S2. Totally differentiated 3T3-L1 cells were cultured for 24 h in a medium containing D-allulose (1-10-20 mM) and treated for 24 h only with the PA vehicle (see material and methods section for more details). Cells treated with the PA vehicle alone were used as controls. (A and B) C/EBP-β and PPAR-γ protein levels; the densitometry results are reported as fold change compared to controls, and the intensity values were normalized to the corresponding value of β-actin. Bands were cropped from original western blot image for illustration purposes; the uncropped images are available in the supplementary material. (C,D and E) *FABP4, SREBP-1* and *FASN* mRNA levels; data are expressed as $2^{-\Delta\Delta Ct}$ and normalized to controls; 18S rRNA was used as housekeeping gene. (F) PPAR-γ protein levels in 3T3-L1 cells cultured with a medium containing three different concentrations of glucose (5-15-25 mM) simulating the glucose amount present in D-Allulose treatments (see material and methods section for more details); the densitometry results are reported as fold change compared to controls, and the intensity values were normalized to the corresponding value of β-actin. All data are expressed as mean ± SD of three independent experiments (n = 3) each performed in triplicate. Means with the same letter are not significantly different from each other ($p > 0.05$)

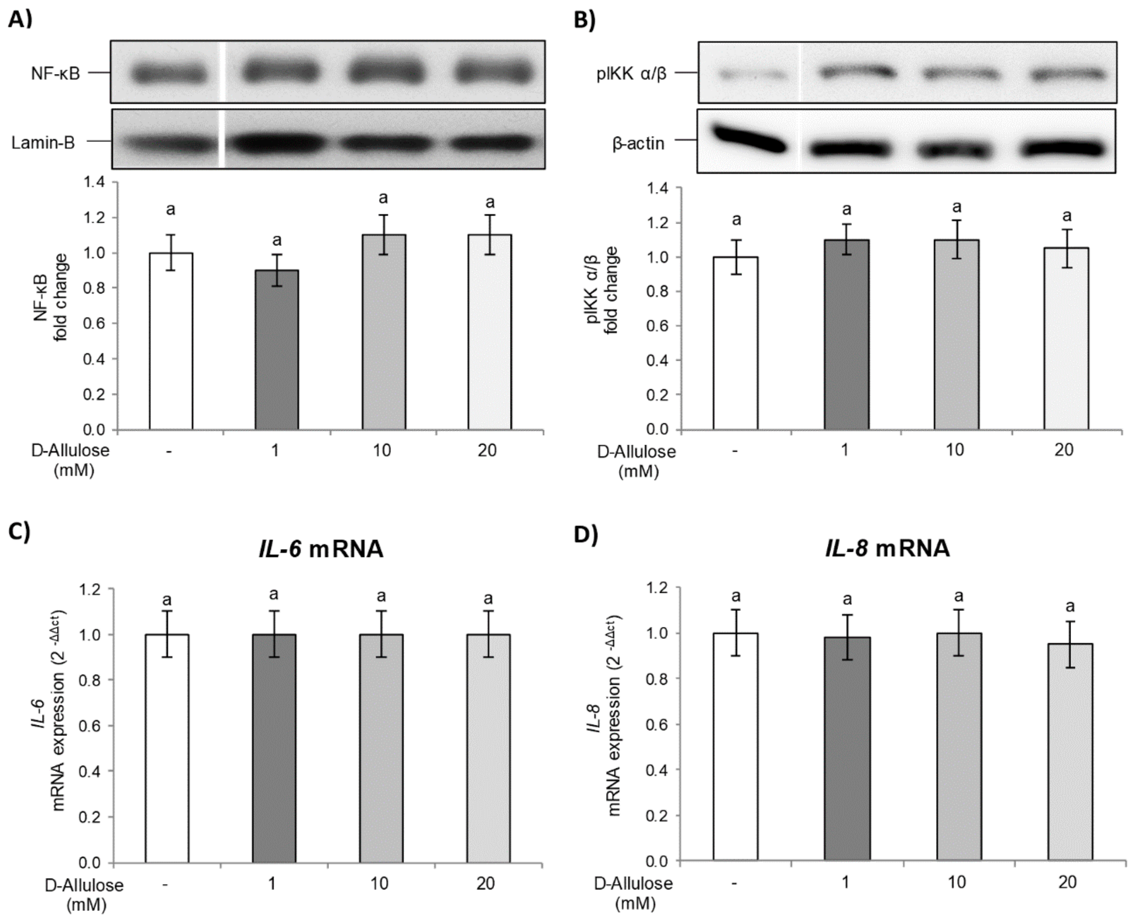

Figure S3. Totally differentiated 3T3-L1 cells were cultured for 24 h in a medium containing D-allulose (1-10-20 mM) and treated for 24 h only with the PA vehicle (see material and methods section for more details). Cells treated with the PA vehicle alone were used as controls. (A and B) Nuclear NF-κB and cytoplasmatic pIKK α/β protein levels; the densitometry results are reported as fold change compared to controls, and the intensity values of NF-κB and pIKK α/β proteins were normalized to the corresponding value of lamin-B and β-actin respectively. Bands were cropped from original western blot image for illustration purposes; the uncropped images are available in the supplementary material. (C and D) *IL-6* and *IL-8* mRNA expression; data are expressed as $2^{-\Delta\Delta Ct}$ and normalized to controls; 18S rRNA was used as housekeeping gene. All data are expressed as mean ± SD of three independent experiments (n = 3) each performed in triplicate. Means with the same letter are not significantly different from each other (p >0.05).

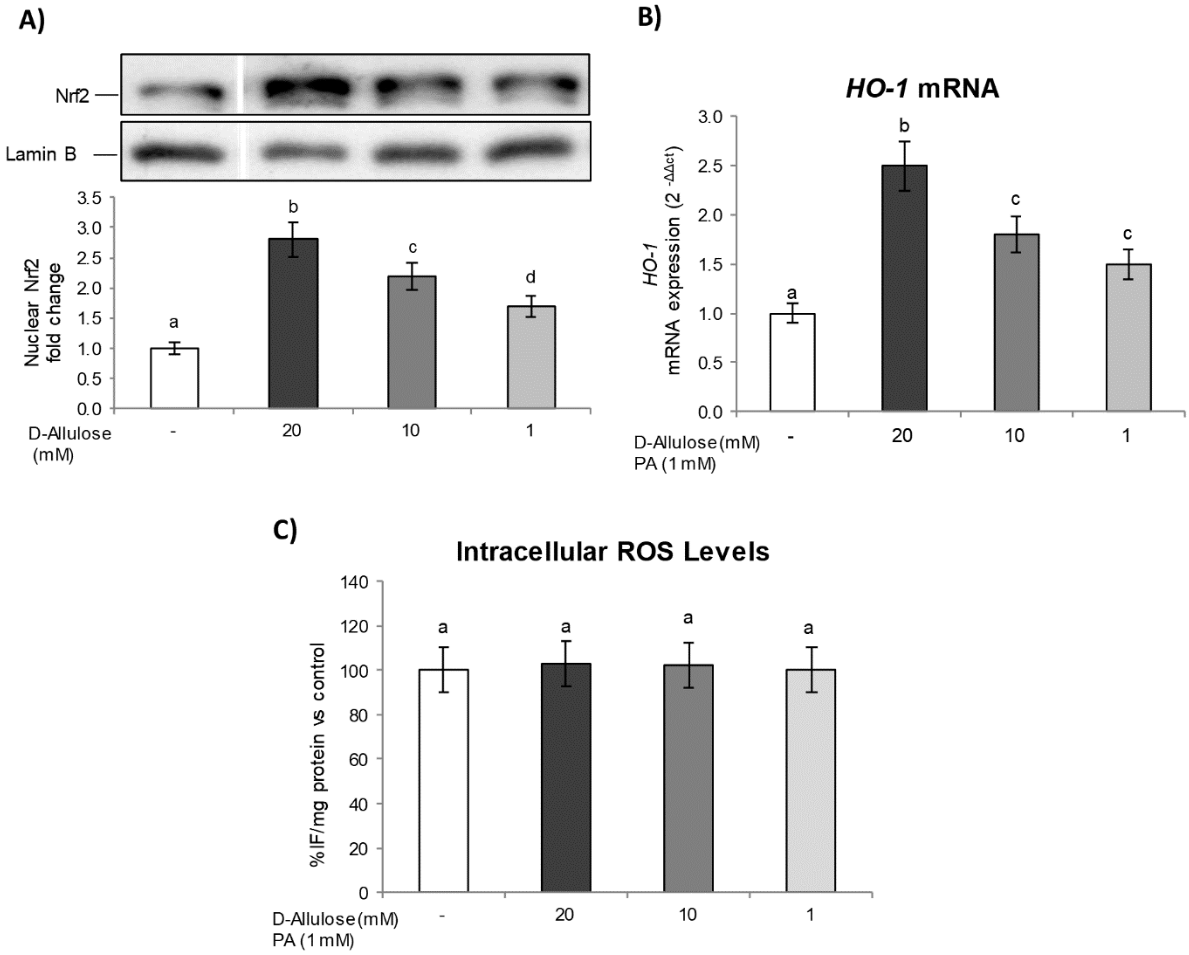

Figure S4. Totally differentiated 3T3-L1 cells were cultured for 24 h in a medium containing D-allulose (1-10-20 mM) and treated for 24 h only with the PA vehicle (see material and methods section for more details). Cells treated with the PA vehicle alone were used as controls. (A) Intracellular ROS levels; the results are reported as % of fluorescence intensity/mg of proteins against control. (B) Modulation of Nrf2 nuclear level; the densitometry results are reported as fold change compared to controls, and the intensity value of Nrf2 was normalized to the corresponding value of lamin-B. Bands were cropped from original western blot image for illustration purposes; the uncropped images are available in the supplementary material. (C) HO-1 mRNA expression; data are expressed as $2^{-\Delta\Delta Ct}$ and normalized to controls; 18S rRNA was used as housekeeping gene. All data are expressed as mean ± SD of three independent experiments (n = 3) each performed in triplicate. Means with the same letter are not significantly different from each other (p >0.05).

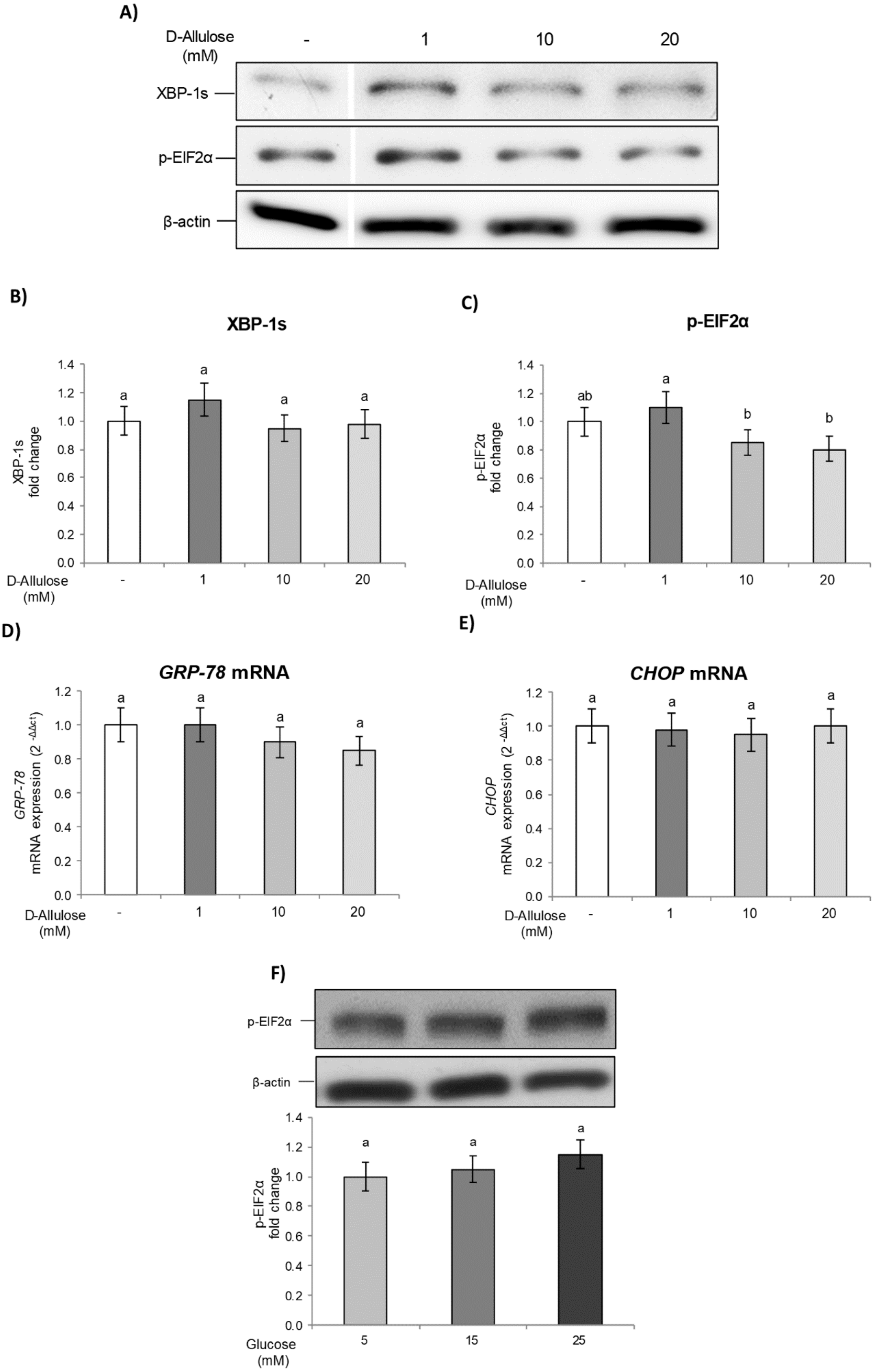
A)
D-Allulose (mM)
-
1
10
20
XBP-1s
p-EIF2α
β-actin
B)
XBP-1s
XBP-1s fold change
a
a
a
a
D-Allulose (mM)
-
1
10
20
C)
p-EIF2α
p-EIF2α fold change
ab
a
b
b
D-Allulose (mM)
-
1
10
20
D)
GRP-78 mRNA
GRP-78 mRNA expression (2 -ΔΔct)
a
a
a
a
D-Allulose (mM)
-
1
10
20
E)
CHOP mRNA
CHOP mRNA expression (2 -ΔΔct)
a
a
a
a
D-Allulose (mM)
-
1
10
20
F)
p-EIF2α
β-actin
p-EIF2α fold change
a
a
a
Glucose (mM)
5
15
25

Figure S5. Totally differentiated 3T3-L1 cells were cultured for 24 h in a medium containing D-allulose (1-10-20 mM) treated for 24 h only with the PA vehicle (see material and methods section for more details). Cells treated with the PA vehicle alone were used as controls. XBP-1s (A, B) and p-EIF2α (A, C) protein levels; the densitometry results are reported as fold change compared to CTR, and the intensity values were normalized to the corresponding value of β-actin. Bands were cropped from original western blot image for illustration purposes; the uncropped images are available in the supplementary material. (D) *GRP-78* and (E) *CHOP* mRNA expression was analyzed by real time PCR and data are expressed as $2^{-\Delta\Delta Ct}$ and normalized to controls; 18S rRNA was used as housekeeping gene. (F) p-EIF2α protein levels in 3T3-L1 cells cultured with a medium containing three different concentrations of glucose (5-15-25 mM) simulating the glucose amount present in D-allulose treatments (see material and methods section for more details); the densitometry results are reported as fold change compared to controls, and the intensity values were normalized to the corresponding value of β-actin. All data are expressed as mean ± SD of three independent experiments (n = 3) each performed in triplicate. Means with the same letter are not significantly different from each other ($p > 0.05$).
