# Supplementary material for: D-Allulose Reduces Hypertrophy and Endoplasmic Reticulum Stress Induced by Palmitic Acid in Murine 3T3-L1 Adipocytes
Source: Int J Mol Sci. 2024 Apr 5;25(7):4059. doi: 10.3390/ijms25074059 (PMC11012259; doi:10.3390/ijms25074059)
Supplement: Supplementary file 1 [file ijms-25-04059-s001.zip › Supplementary Material 2 - Original Images for Blots.pdf]

Supplementary material

D-Allulose Reduces Hypertrophy and Endoplasmic Reticulum Stress Induced by Palmitic Acid in Murine 3T3-L1 Adipocytes

Maria Sofia Molonia [1,2], Federica Lina Salamone [1], Antonio Speciale [1,*], Antonella Saija [1,†] and Francesco Cimino [1,†]

Original images for blots of figure 2A and S2A: CEBP/β and β actin. In the black boxes the parts of the images used in figure 2A. In the red boxes the parts of the images used in figure S2A.

C/EBP-β

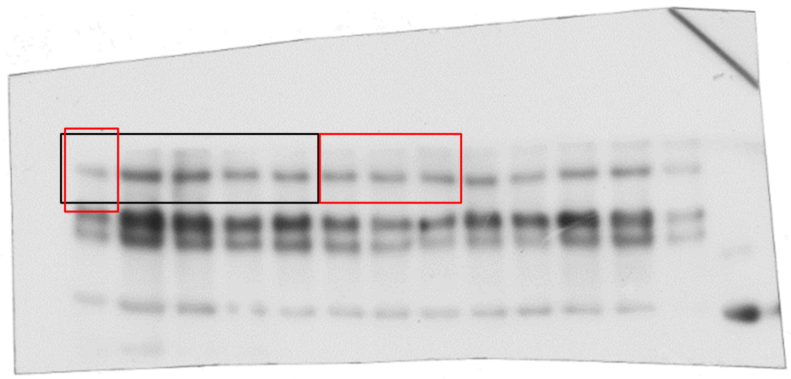

β-actin

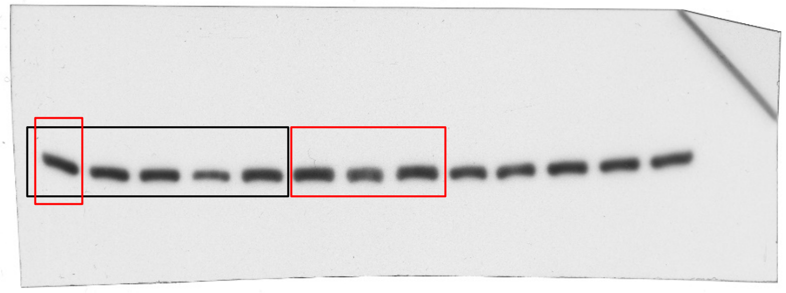

Sample loading order: 1° **CTR** – 2° **PA 1 mM**– 3° **D-Allulose 1 mM + PA –** 4° **D-Allulose 10 mM + PA** – 5° **D-Allulose 20 mM + PA** – 6° **D-Allulose 1 mM –** 7° **D-Allulose 10 mM-** 8° **D-Allulose 20 mM -** other samples

Original images for blots of figure 2B and S2B: **PPARγ and β actin.** In the black boxes the parts of the images used in figure 2B. In the red boxes the parts of the images used in figure S2B.

PPARγ

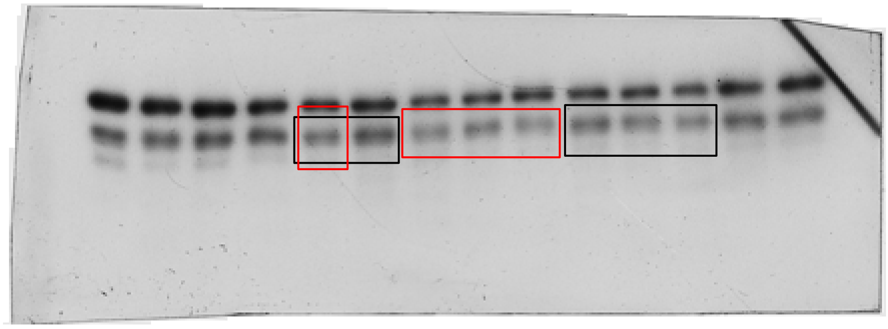

β-actin

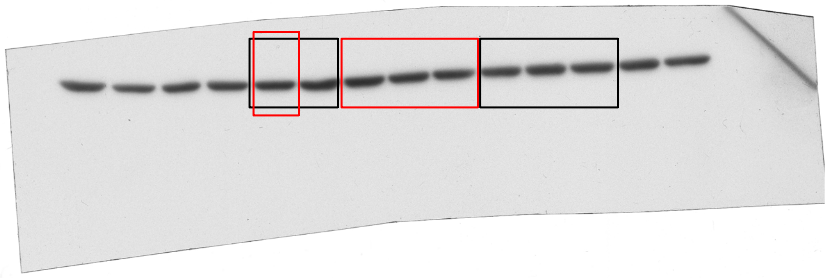

Sample loading order: other samples - 5° **CTR** – 6° **PA 1 mM**–7° **D-Allulose 1 mM –** 8° **D-Allulose 10 mM** – 9° **D-Allulose 20 mM** – 10° **D-Allulose 1 mM + PA –** 11° **D-Allulose 10 mM + PA –** 12 ° **D-Allulose 20 mM + PA -** other samples

Original images for blots of figure 3A and S3A: NF-κB and Lamin B. In the black boxes the parts of the images used in figure 3A. In the red boxes the parts of the images used in figure S3A.

NF-κB

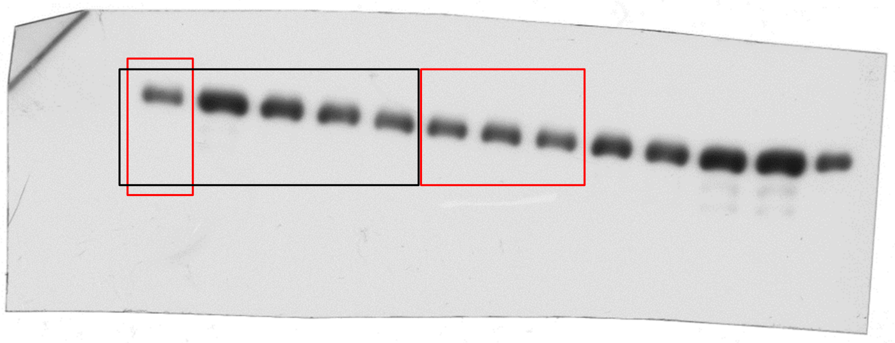

Lamin B

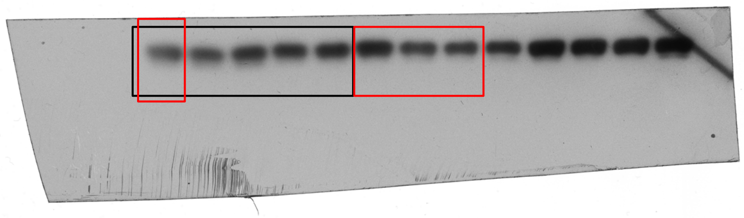

Sample loading order: 1° **CTR** – 2° **PA 1 mM**– 3° **D-Allulose 1 mM + PA –** 4° **D-Allulose 10 mM + PA** – 5° **D-Allulose 20 mM + PA** – 6° **D-Allulose 1 mM –** 7° **D-Allulose 10 mM-** 8° **D-Allulose 20 mM -** other samples

Original images for blots of figure 3B and S3B: pIKK α/β and β actin. In the black boxes the parts of the images used in figure 3B. In the red boxes the parts of the images used in figure S3B.

pIKK α/β

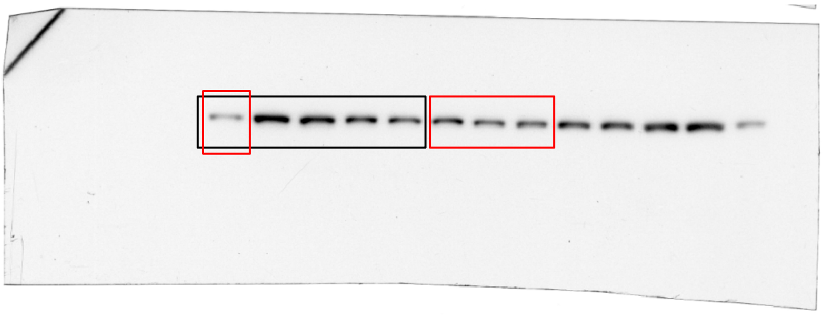

β actin

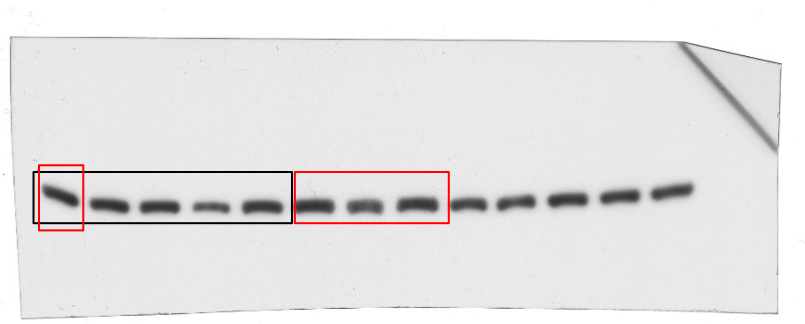

Sample loading order: 1° **CTR** – 2° **PA 1 mM**– 3° **D-Allulose 1 mM + PA – **4° **D-Allulose 10 mM + PA** – 5° **D-Allulose 20 mM + PA** – 6° **D-Allulose 1 mM –** 7° **D-Allulose 10 mM-** 8° **D-Allulose 20 mM -** other samples

Original images for blots of figure 5A and S5A: **XBP-1s and β actin.** In the black boxes the parts of the images used in figure 5A. In the red boxes the parts of the images used in figure S5A.

XBP-1s

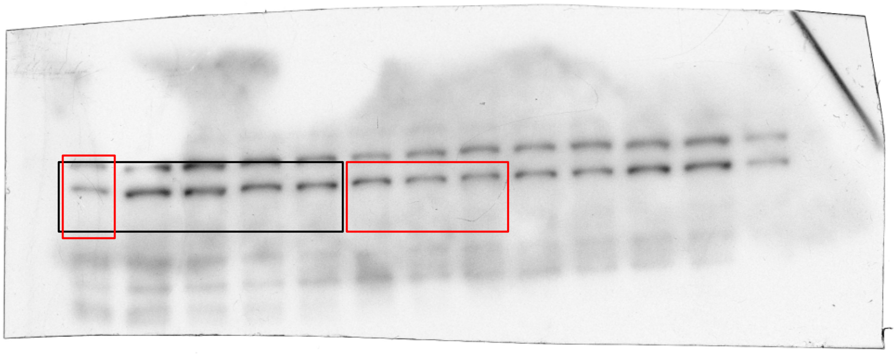

β-actin

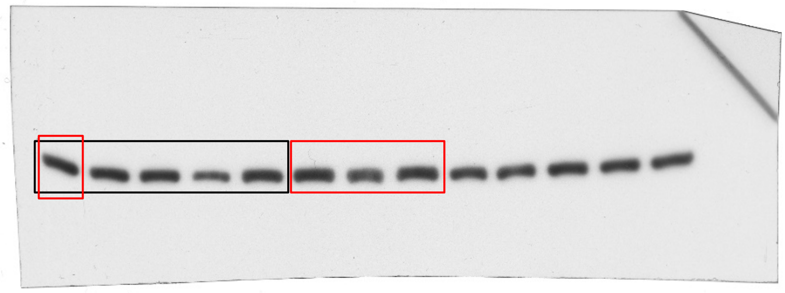

Sample loading order: 1° **CTR** – 2° **PA 1 mM**– 3° **D-Allulose 1 mM + PA –** 4° **D-Allulose 10 mM + PA** – 5° **D-Allulose 20 mM + PA** – 6° **D-Allulose 1 mM –** 7° **D-Allulose 10 mM-** 8° **D-Allulose 20 mM -** other samples

Original images for blots of figure 5B and S5B: **pEIF-2α and β-actin.** In the black boxes the parts of the images used in figure 6B. In the red boxes the parts of the images used in figure S6B.

pEIF-2α

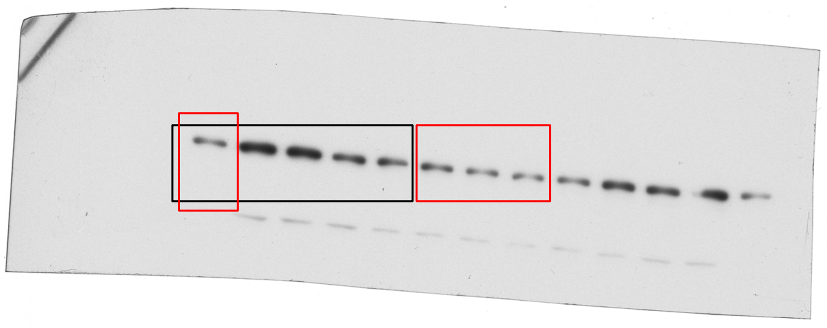

β-actin

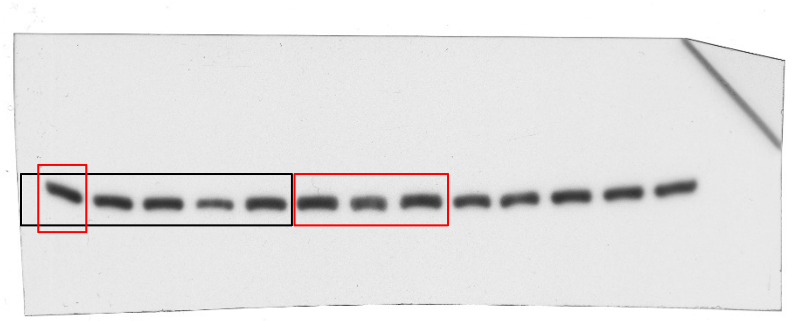

Sample loading order: 1° **CTR** – 2° **PA 1 mM**– 3° **D-Allulose 1 mM + PA –** 4° **D-Allulose 10 mM + PA** – 5° **D-Allulose 20 mM + PA** – 6° **D-Allulose 1 mM –** 7° **D-Allulose 10 mM-** 8° **D-Allulose 20 mM -** other samples

Original images for blots of figure 6A: **PPAR-γ and β actin.** In the black box the parts of the image used in figure 6A.

PPAR-γ

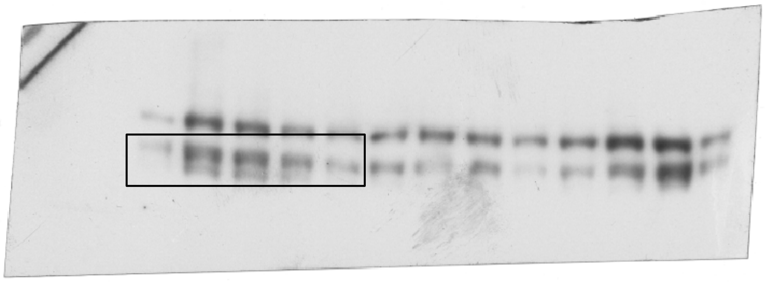

β-actin

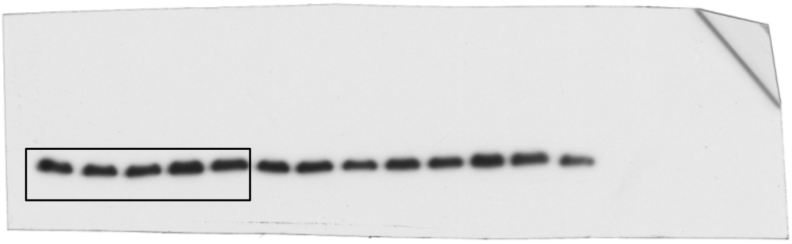

Sample loading order: 1° **CTR** – 2° **PA 1 mM**– 3° **PA + D-Allulose 1 mM –** 4° **PA + D-Allulose 10 mM** – 5° **PA + D-Allulose 20 mM -** other samples

Original images for blots of figure 6B: **NF-κB and Lamin B.** In the black box the part of the image used in figure 6B.

NF-κB

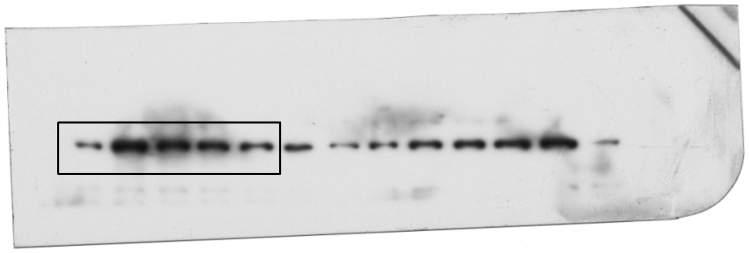

Lamin B

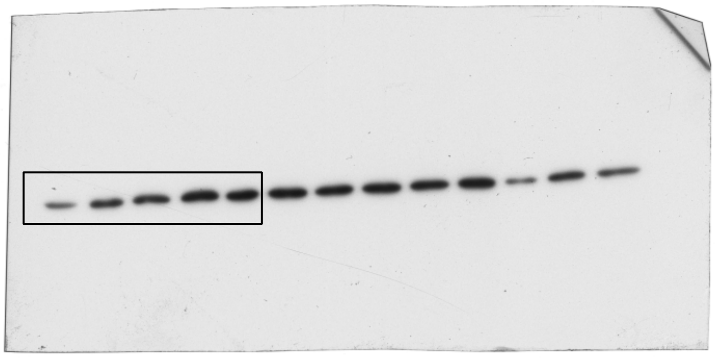

Sample loading order: 1° **CTR** – 2° **PA 1 mM**– 3° **PA + D-Allulose 1 mM –** 4° **PA + D-Allulose 10 mM** – 5° **PA + D-Allulose 20 mM** –other samples

Original images for blots of figure 6C: **pEIF-2α and β-actin.** In the black box the part of the image used in figure 6C.

pEIF-2α

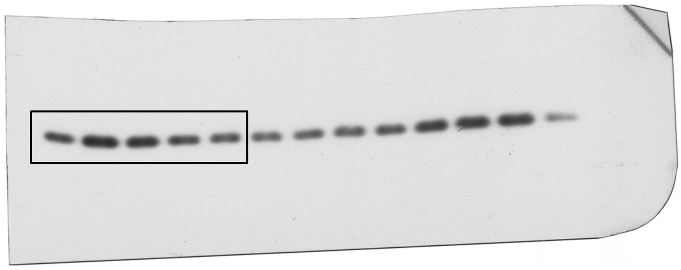

β-actin

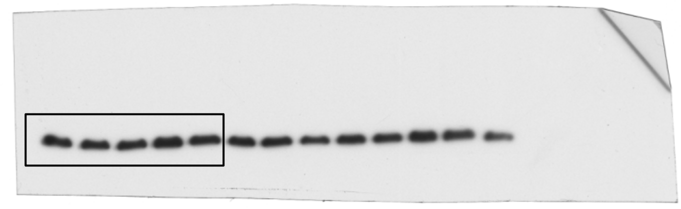

Sample loading order: 1° **CTR** – 2° **PA 1 mM**– 3° **PA + D-Allulose 1 mM –** 4° **PA + D-Allulose 10 mM** – 5° **PA + D-Allulose 20 mM** –other samples

The correct migration of each protein of interest into the gels was verified by the use of molecular weight markers comparing membranes and films.
